# Family living sets the stage for cooperative breeding and ecological resilience in birds

**DOI:** 10.1371/journal.pbio.2000483

**Published:** 2017-06-21

**Authors:** Michael Griesser, Szymon M. Drobniak, Shinichi Nakagawa, Carlos A. Botero

**Affiliations:** 1 Department of Anthropology, University of Zurich, Zurich, Switzerland; 2 Department of Ecology, Swedish University of Agricultural Sciences, Uppsala, Sweden; 3 Institute of Environmental Sciences, Jagiellonian University, Kraków, Poland; 4 Evolution & Ecology Research Centre and School of Biological, Earth and Environmental Sciences, University of New South Wales, Sydney, Australia; 5 Department of Biology, Washington University in St. Louis, St. Louis, Missouri, United States of America; Ecole Normale Superieure, France

## Abstract

Cooperative breeding is an extreme form of cooperation that evolved in a range of lineages, including arthropods, fish, birds, and mammals. Although cooperative breeding in birds is widespread and well-studied, the conditions that favored its evolution are still unclear. Based on phylogenetic comparative analyses on 3,005 bird species, we demonstrate here that family living acted as an essential stepping stone in the evolution of cooperative breeding in the vast majority of species. First, families formed by prolonging parent–offspring associations beyond nutritional independency, and second, retained offspring began helping at the nest. These findings suggest that assessment of the conditions that favor the evolution of cooperative breeding can be confounded if this process is not considered to include 2 steps. Specifically, phylogenetic linear mixed models show that the formation of families was associated with more productive and seasonal environments, where prolonged parent–offspring associations are likely to be less costly. However, our data show that the subsequent evolution of cooperative breeding was instead linked to environments with variable productivity, where helpers at the nest can buffer reproductive failure in harsh years. The proposed 2-step framework helps resolve current disagreements about the role of environmental forces in the evolution of cooperative breeding and better explains the geographic distribution of this trait. Many geographic hotspots of cooperative breeding have experienced a historical decline in productivity, suggesting that a higher proportion of family-living species could have been able to avoid extinction under harshening conditions through the evolution of cooperative breeding. These findings underscore the importance of considering the potentially different factors that drive different steps in the evolution of complex adaptations.

## Author summary

Cooperative breeding is a common form of cooperation in which individuals help raise conspecific offspring that are not their own. It has evolved in a range of lineages, including arthropods, fish, birds, and mammals. In birds, cooperative breeding is widespread and well-studied; however, the conditions that favored its evolution are still unclear. Based on an analysis of 3,005 bird species, we show that the evolution of this social system required 2 transitions. First, families formed by prolonging parent–offspring associations, and second, retained offspring began helping at the nest. We then show that the formation of families is associated with more productive and seasonal environments and that the subsequent evolution of cooperative breeding is linked to an increase in the variability of environmental productivity. These findings are consistent with patterns in insects and mammals (including humans) and clarify current disagreements on the role of environmental forces in the evolution of cooperation.

## Introduction

Cooperative breeding is an extreme form of cooperation that occurs when individuals help raise conspecific offspring that are not their own [1], often while temporarily foregoing their own reproduction [2,3]. This common form of cooperation has intrigued evolutionary biologists since Darwin [4] and is thought to have evolved multiple times in a range of lineages, including insects, fish, birds, and mammals, usually as a product of kin selection [5]. Even though life history and ecological correlates of cooperative breeding have been particularly well studied in birds [6–9], large-scale comparative analyses in this group have yielded contradictory findings [6,7,9,10]. Thus, the conditions that favor the evolution of cooperative breeding are currently unclear [2].

Earlier theoretical work suggested that delayed dispersal (i.e., family formation) is a critical step in the evolution of cooperative breeding [3,11,12], reflecting the fact that helping at the nest in birds is overwhelmingly kin-based [13–15]. These studies proposed that family living arises when parents can afford to invest in offspring beyond independence, which is more likely in long-lived species [12,16] and in stable and productive environments that allow for a prolonged association of offspring with their parents [17,18]. However, subsequent work has generally overlooked that many bird species live in families that do not breed cooperatively [14]. Consequently, prior comparative analyses have investigated the evolution of cooperative breeding by contrasting cooperative and noncooperative species [7,9,10,19–23] and have provided equivocal predictions about the occurrence of cooperative breeding. For example, these studies suggest that cooperative breeding may be favored either when living in saturated habitats with a slow turnover in breeding opportunities (i.e., stable environments with a long mean growing season [MGS] [3,7,10,11,24]) or when living in unpredictable environments, where helpers at the nest can buffer reproductive failure in harsh years (i.e., high degree of unpredictability [3,6,9,23,25]). Under both of these hypotheses, cooperative breeding is predicted to evolve preferentially in species with a high survival probability [10], because high survival increases the time offspring have to queue for breeding opportunities, increases habitat saturation, and enhances opportunities to act as helper at the nest [26].

Here, we test the hypothesis that the evolution of cooperative breeding from a noncooperative ancestor may have involved 2 distinct transitions: one to a continued parent–offspring association beyond the period when offspring are actively provisioned by their parents (i.e., the formation of families [14]) and a subsequent one to the evolution of helping at the nest. We posit that, by considering only 1 transition from noncooperative breeding to cooperative breeding, prior studies may have obscured the role of potential ecological and life history drivers because the factors that promote family living may have been inadvertently confused with those that promote helping at the nest [14,18]. Thus, a more nuanced understanding of the evolutionary steps through which cooperative breeding arose may help clarify the current debate on the conditions favoring its evolution [6,7,9,10].

**Fig 1:**
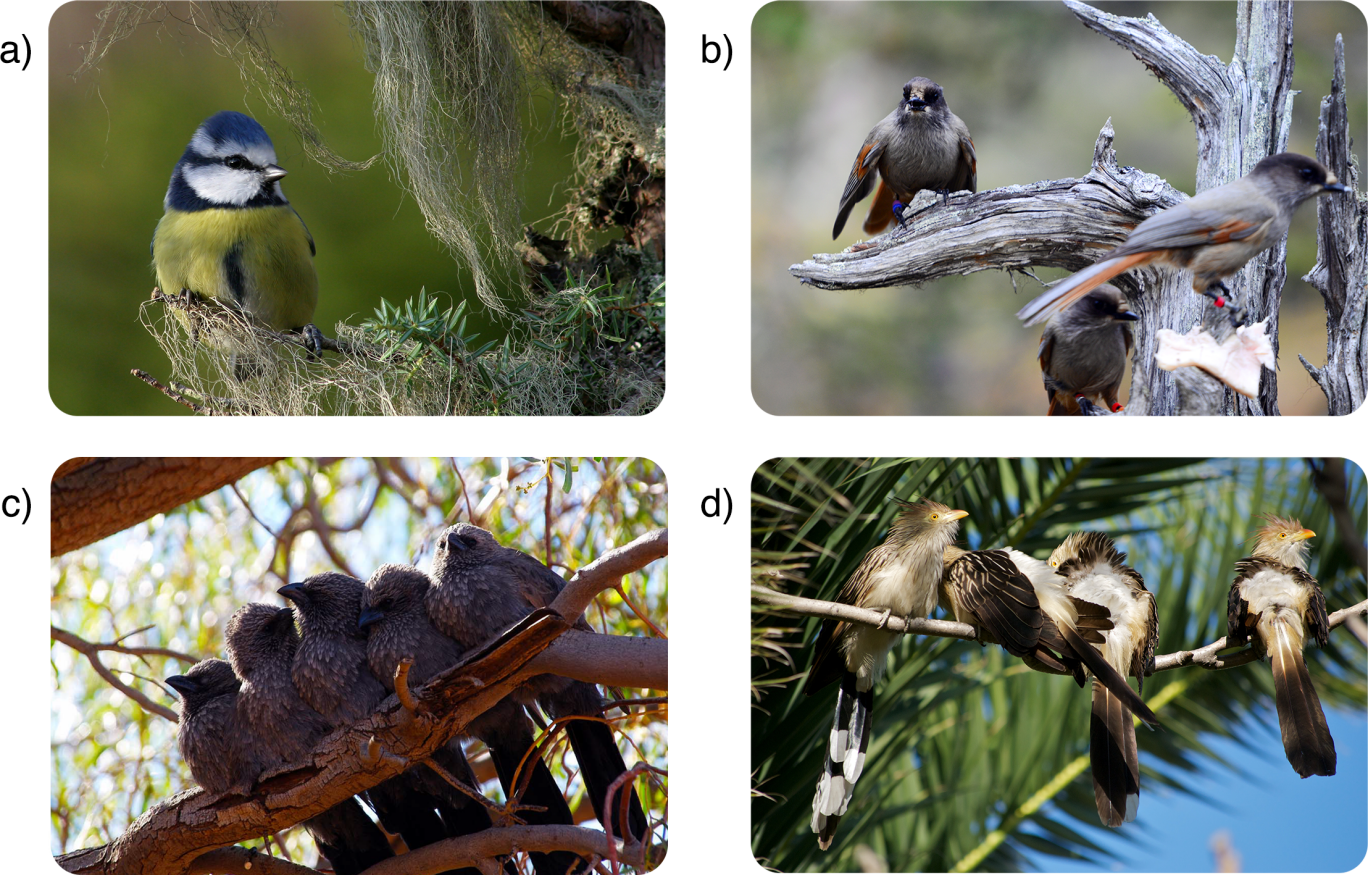
Avian social systems. Social systems include non-family-living species (55% in our data set, e.g., the blue tit *Parus caeruleus* [a]), in which parent–offspring associations do not extend beyond nutritional independence and individuals that do not engage in cooperative breeding; family-living species (31% in our data set, e.g., the Siberian jay *Perisoreus infaustus* [b]; see also S1 Fig), in which offspring remain with their parents beyond nutritional independence but do not aid in the rearing of future broods; and cooperative breeding species (13% in our data set, e.g., the apostlebird *Struthidea cinerea* [c]), in which offspring remain with their parents beyond nutritional independence and help them in subsequent breeding attempts or engage in redirected helping at nests of relatives. In a small number of species (1% in our data set), e.g., in the guira cuckoo *Guira* (d), cooperative breeding primarily involves nonrelatives (“non-kin cooperatively breeding species”). (a) *Image credit*: *Per Harald Olsen/NTNU*. (b) *Image credit*: *Michael Griesser*. (c) *Image credit*: *Michael Griesser*. (d) *Image credit*: *Beatrice Murch*.

We took advantage of the extensive natural history data on the social life of birds (*N* = 3,005 terrestrial species, including species from all major orders and bioregions, see S1 Table for details) to categorize species into 1 of 4 social systems. The 3 most common systems are: (i) “non-family-living species,” in which parent–offspring associations do not extend beyond nutritional independence and individuals do not engage in cooperative breeding (55% in our data set; Fig 1A); (ii) "family-living species,” in which offspring remain with their parents beyond nutritional independence but the retained offspring do not assist their parents in rearing activities [14] (this includes species with both biparental and uniparental brood care; 31% in our data set; Fig 1B); and (iii) “cooperatively breeding species,” in which offspring remain with their parents beyond nutritional independence and help them in subsequent breeding attempts or engage in redirected helping at nests of relatives (13% in our data set; Fig 1C). Family-living and cooperatively breeding species differ not only in terms of helping at the nest but also in that offspring in 91% of family-living species disperse before the onset of the next breeding season. Finally, the fourth social system involves a very limited number of bird species that exhibit helping at the nest among unrelated individuals [8,13] (“non-kin cooperatively breeding species”; 1% in our data set; Fig 1D).

To test the suitability of the proposed 2-step model for the evolution of cooperative breeding, we first estimated the relative rates of evolutionary transitions among different avian social systems and investigated whether family living was a necessary precursor for the evolution of cooperative breeding. We then evaluated the ecoclimatic correlates of each social system to gain insight into the potential pressures of selection that drove each of these evolutionary transitions, with a particular focus on distinguishing the conditions that promoted the formation of family groups from those that favored the evolution of cooperative breeding.

## Results

**Fig 2:**
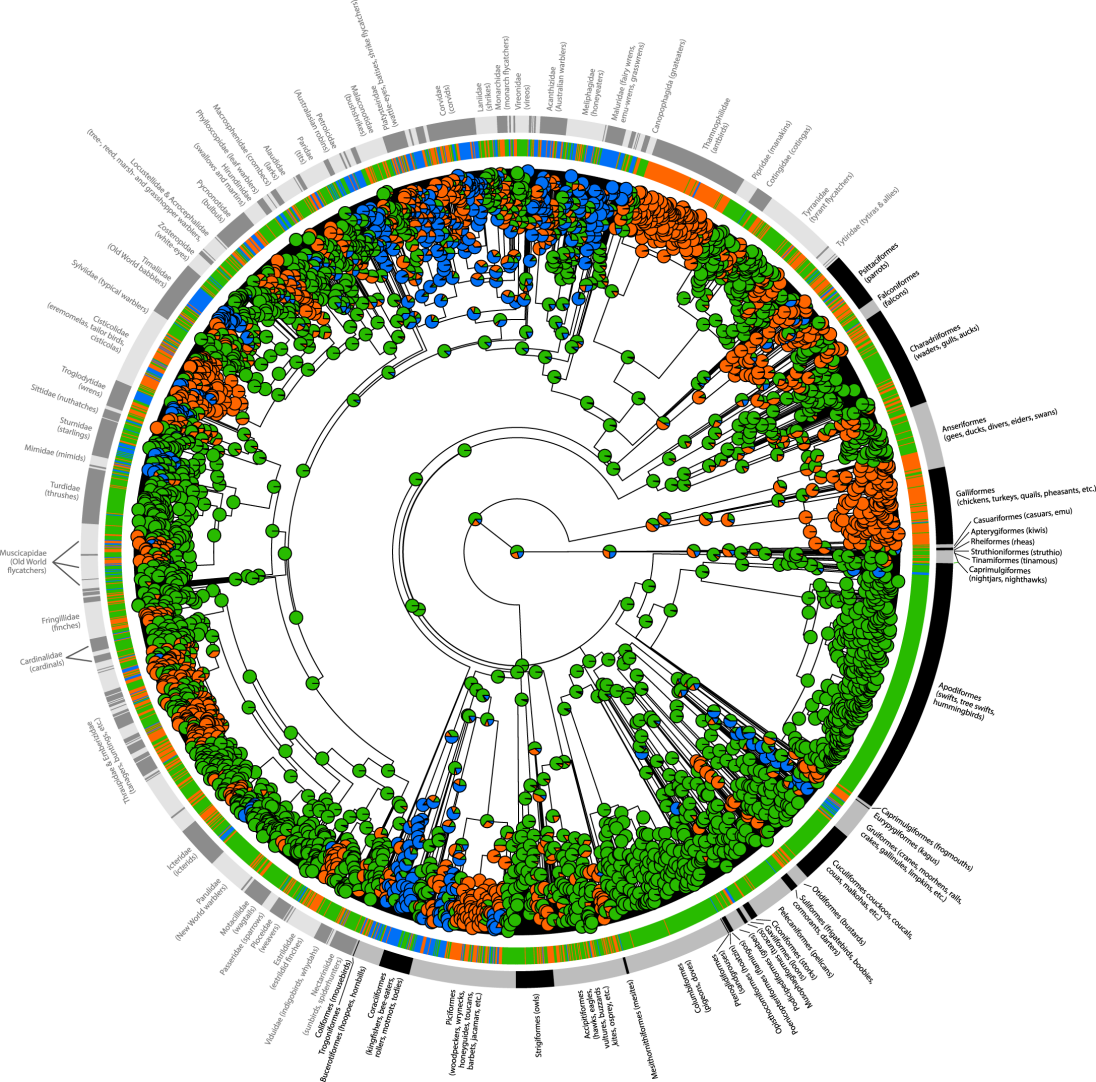
Ancestral state reconstruction (based on maximum likelihood) and estimated evolutionary transitions of bird social system (*N* = 2,968 species). Pie charts plotted at each node represent the estimated posterior proportion of the 3 social systems: non-family living (green), family living (orange), and cooperative breeding families (blue).

**Fig 3:**
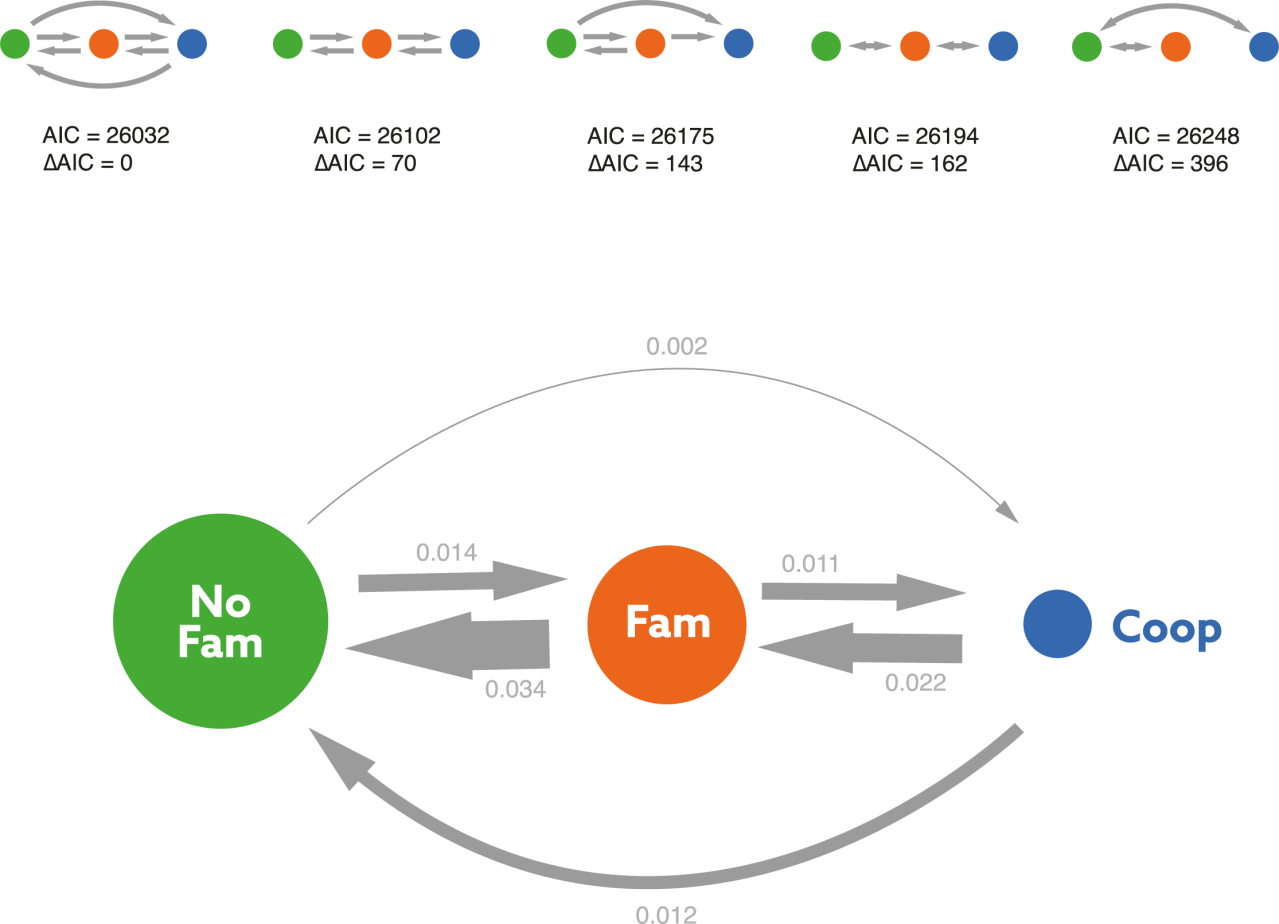
**Estimated transition rates of the best-fitting model (a) and statistical evaluation of the different transition models of the evolution of avian social systems (b).** In the best-fitting transition model, arrow thickness is proportional to the estimated transition rates, and the size of the circles is proportional to the relative abundance of the 3 social systems among the species in the sample. No Fam = non-family living; Fam = family living; Coop = cooperative breeding families. Directions of the arrows indicate modelled transitions: a single arrow between 2 states pointing in both directions reflects transition rates constrained to be equal, a single arrow pointing in 1 direction reflects transitions only in 1 direction, and 2 arrows between states reflects unconstrained transition rates. AIC = Akaika information criterion.

Given the rarity of non-kin helping (see above), we began our analyses by focusing on the 3 major avian social systems (i.e., non-family-living, family-living, and cooperatively breeding families). Based on a recent class-wide phylogeny [27] and a model of discrete trait evolution [28], we estimated evolutionary transitions between these social systems and confirmed that the ancestral social system in birds was very likely to be non-family living (Fig 2). Transitions between non-family living and family living, as well as those between family living and cooperative breeding, were common (i.e., transition rates range from 0.01 to 0.04; Fig 3). Importantly, however, direct transitions from non-family living to cooperative breeding were exceedingly rare (transition rate = 0.002; Fig 3). Including non-kin cooperatively breeding species in the analysis showed that this system mostly arose from non-family-living species but does not have an evolutionary link to family-based cooperative breeding (S2 Fig). These results strongly suggest that the evolution of family living was a pivotal precondition for the evolution of cooperative breeding in the majority of birds. Thus, to examine the possible conditions favoring cooperative breeding in birds, we now ask how the predictors of cooperative breeding differ from those of family living.

To investigate the conditions favoring the evolution of family living and cooperative breeding in birds, we used a phylogenetically controlled multinomial generalized linear mixed model [29,30]. Our model explored the effects of putative ecoclimatic, social, and life-history predictors of cooperative breeding explored in previous analyses (i.e., sedentariness [10], stable climatic conditions [3,7], environmental unpredictability [3,6,9,25], nesting modus [31], low annual mortality [10], and altricial offspring that require active food provisioning [32]). We also controlled for the potentially confounding effects of having classified social systems using 3 different sources of information (see Materials and methods). We calculated mean values, predictability indices, and within-year variances for precipitation, temperature, and net primary productivity (NPP) by computing values locally (cell size: 0.5° x 0.5°) and subsequently averaging them across the entire breeding distribution of each species. Because climatic unpredictability during the breeding season is thought to be particularly important for the evolution of cooperative breeding [6], we calculated ecoclimatic correlates both across the entire year and exclusively during the likely breeding season at each location. The duration of avian breeding seasons at a given locality was estimated from the length of the growing season of local plants [33] (see S1 Text).

We used principal component analysis (PCA) to reduce the dimensionality of our original set of 23 continuous predictors because most of them exhibited moderate to strong collinearity. The first 8 principal components (PCs) in this analysis captured 92% of the variance in continuous predictors (S2 Table). Fifteen out of the 19 original environmental variables loaded primarily on the first 2 PCs (PC1 and PC2). PC1, dubbed “variable rainfall among years,” reflects a gradient toward environments where rainfall is higher on average (along with an associated increase in NPP) but more variable among years. PC2, dubbed “mean growing season duration,” reflects a gradient toward longer breeding seasons and more stable temperatures throughout the year. The remaining components capture the residual variance in 1 to 3 variables each, after accounting for correlations with other components (S2 Table). We could not include non-kin cooperatively breeding species as an independent social system in the multinomial analysis because the number of species in this category is too small to derive meaningful estimates of statistical parameters.

**Fig 4:**
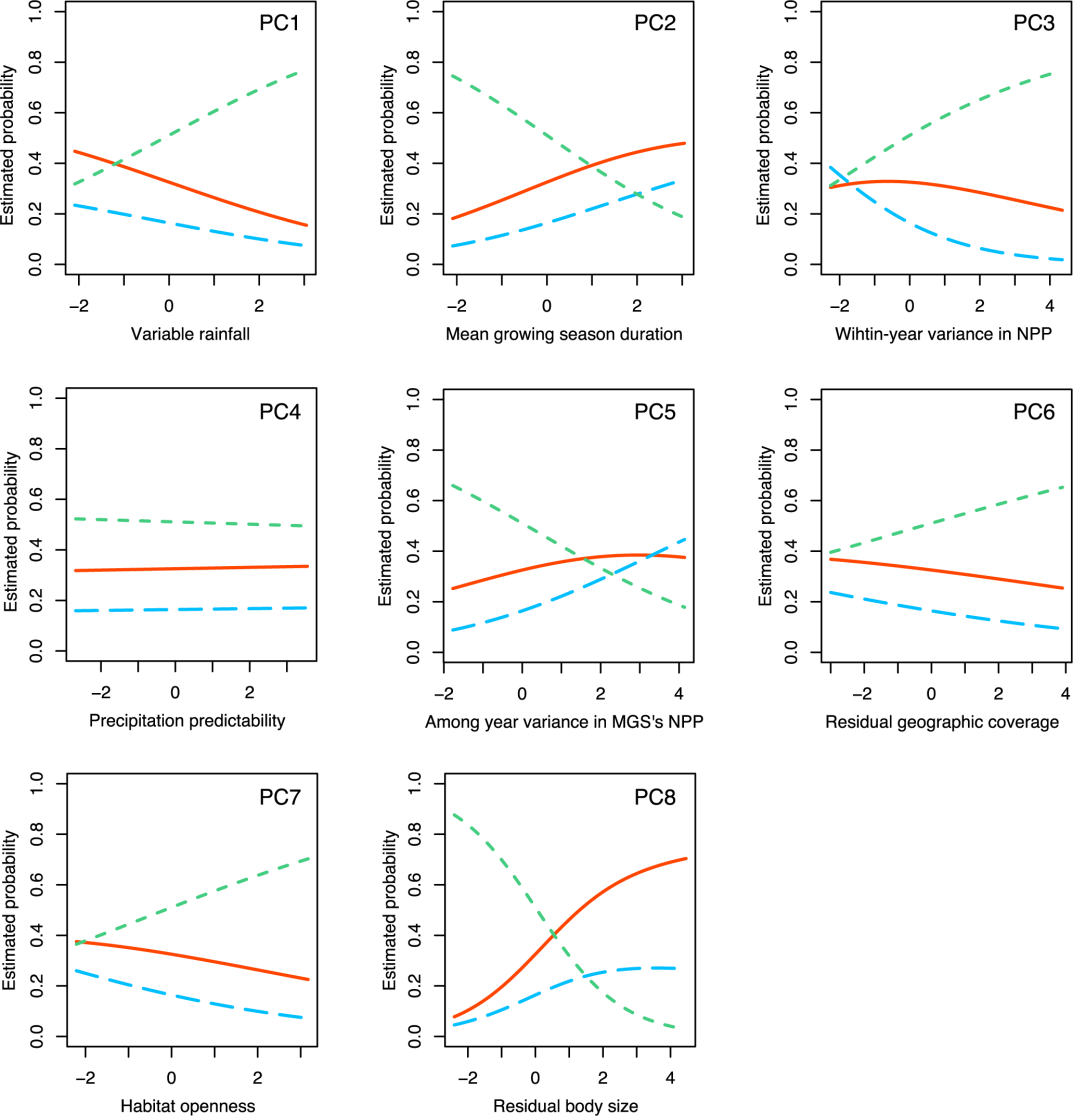
Ecoclimatic and life-history correlates of non-family-living (green dotted line), family-living (orange solid line), and cooperative breeding species (blue dashed line); *N* = 2,968 bird species (excluding cooperative breeding species with non-kin helpers only). Lines reflect the predicted probabilities of occurrence of respective social systems estimated from phylogenetically informed multinomial models (see Table 1). Family-living and cooperative breeding species are associated with locations that have abundant but variable precipitation (PC1), a longer mean growing season (PC2), and a higher among-year variance in net primary productivity (NPP) during the growing season (PC5). Moreover, these species live in denser habitats (PC7) and have a larger body size (PC8). Cooperative breeding species are associated with higher within-year variance in NPP (PC3). MGS, mean growing season.

**Fig 5:**
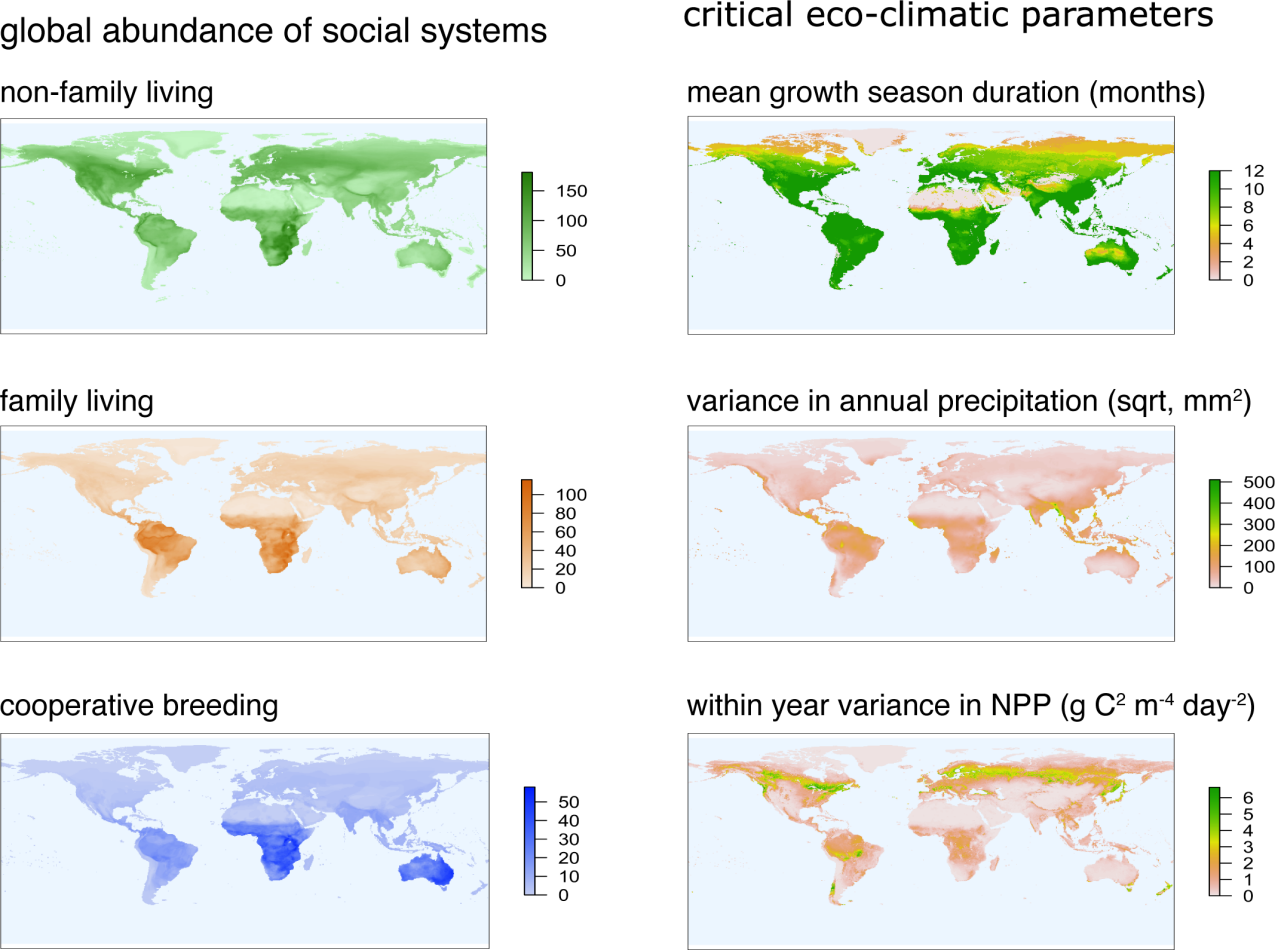
Global abundance of non-family-living, family-living, and cooperative breeding species in birds (number of species per 0.5° x 0.5°) and global patterns of the 3 most influential ecoclimatic parameters (duration of the mean growing season [MGS]; included in PC2), annual variance in precipitation (square-root transformed; included in PC1), and within-year variance in net primary productivity (NPP; included in PC3). Figures were plotted using the letsR package [34]. Abbreviations: sqrt, square-root transformed.

Our multinomial analysis (using family living as the reference level) reveals that non-family living and family living are associated with very different ecoclimatic and life-history variables, while the predictors associated with family living and cooperative breeding are nearly identical (Table 1). Compared to non-family-living species, family-living species have a higher probability of occurrence at localities where rainfall is more abundant and variable (PC1), MGSs are longer (PC2), and the among-year variance in productivity during the growing season is higher (PC5) (Table 1, Figs 4 and 5). Moreover, family-living species are typically larger (PC8, Fig 4), more sedentary, live in denser habitats (PC7, Fig 4) and exhibit a higher degree of food specialization than non-family-living species (Table 1). Thus, many of the ecological conditions currently believed to promote the transition to cooperative breeding are likely to have driven the initial transition to family living instead.

**Table 1:** Multinomial phylogenetically controlled generalized linear mixed models comparing the effect of ecoclimatic and life-history variables on the evolution of non-family-living, family-living, and cooperative breeding species; *N* = 2,968 bird species (excluding cooperative breeding species with non-kin helpers only). Coefficients reflect the results of multinomial phylogenetic regression models with “cooperative families” as the reference category in the analyses and thus not shown per se. Significant factors are highlighted in bold. The principal component analyses (PCAs) resulting in PC1–8 are shown in S2 Table. The factor social system assessment specified whether it was assessed based on the time offspring remained with their parents beyond independence (using 50 days as a threshold to differentiate between non-family-living and family-living species; see [17]), breeding behavior, or social information. MCMC = Markov chain Monte Carlo.

	Family-living species (reference) versus non-family-living species:				Family-living species (reference) versus cooperative breeding species:			
Factor	Mean estimated effect^†^*	95% CI (lower; upper)*	f	p MCMC*	Mean estimated effect^†^*	95% CI (lower; upper)*	f	p MCMC*
Intercept	0.5	**–**0.75; 1.88	0	0.46	**–**0.76	**–**1.83; 0.46	0	0.18
Variable rainfall (PC1)	**0.42**	**0.2; 0.62**	**1**	**0.0011**	**–**0.02	**–**0.31; 0.29	0	0.91
Mean growing season duration (PC2)	**–0.51**	**–0.73; –0.3**	**1**	**0.0011**	0.12	**–**0.19; 0.44	0	0.47
Within-year variance in productivity (PC3)	**0.22**	**0.05; 0.41**	**1**	**0.024**	**–0.43**	**–0.65; –0.13**	**1**	**0.0011**
Precipitation predictability (PC4)	**–**0.02	**–**0.21; 0.19	0	0.83	0	**–**0.27; 0.3	0	0.95
Among-year variance in MGS's NPP (PC5)	**–0.32**	**–0.51; –0.12**	**1**	**0.0043**	0.23	**–**0.02; 0.48	0	0.063
Residual geographic range (PC6)	0.14	**–**0.04; 0.32	0	0.13	**–**0.09	**–**0.32; 0.16	0	0.52
Residual habitat openness (PC7)	**0.24**	**0.02; 0.45**	**1**	**0.034**	**–**0.16	**–**0.47; 0.08	0	0.25
Residual body size (PC8)	**–0.92**	**–1.32; –0.5**	**1**	**0.0011**	**–**0.07	**–**0.52; 0.35	0	0.72
Chick development modus (altricial versus precocial)^‡^	**–**0.51	**–**1.68; 0.68	0	0.43	**–**0.61	**–**1.91; 0.42	0	0.33
Food specialization (generalist versus specialist) ^‡^	**–0.59**	**–1.07; –0.22**	**1**	**0.0086**	0.48	**–**0.05; 1.07	0	0.1
Sedentariness (sedentary versus migratory) ^‡^	**–0.94**	**–1.45; –0.4**	**1**	**0.0011**	0.34	**–**0.46; 1.22	0	0.44
Nest type (cavity versus open nesting) ^‡^	**–**0.19	**–**0.82; 0.44	0	0.51	**–**0.36	**–**1.09; 0.47	0	0.35
Social system assessment—breeding behavior	**–1.08**	**–1.93; –0.19**	**1**	**0.013**	**4.2**	**3.46; 4.96**	**1**	**0.0011**
Social system assessment—social information	0	-0.37; 0.42	0	0.96	**–3.78**	**–4.36; –3.09**	**1**	**0.0011**

^‡^ Reference level is the first category in these lists

*Average over all 50 models

**Abbreviations:** f, frequency of trees for which MCMC p-values < 0.05 (*N* = 50 randomly selected phylogenetic trees); MGS, mean growing season, NPP, net primary productivity.

In contrast, our analyses revealed very few differences in the predictors of cooperative breeding and family living. An important difference is that cooperatively breeding species are more likely to occupy environments with a high within year variability in environmental productivity, whereas family-living species are more common in localities where the within year variance in productivity is intermediate (PC3, Figs 4 and 5). This result suggests that helping at the nest evolved where family-living species faced environments with more variable productivity, supporting the hard life hypothesis [25] and the environmental unpredictability hypothesis [6,9].

Earlier studies suggested that prolonged parental investment [14,16] and cooperative breeding [35] are associated with a high survival probability. Given that survival is poorly studied in most species, we included longevity instead as its proxy in our models (available for *N* = 1,023 species). However, this model did not reveal any additional effects on the distribution of social systems (S3 Table), although we note that longevity can be a poor surrogate for annual survival. We also note that the subsample of birds for which longevity is known is biased toward temperate, non-family-living species and that estimates of longevity are highly influenced by sampling effort [36].

## Discussion

Overall, our results help unravel the potential sequence of evolutionary steps in the evolution of cooperative breeding and provide a clearer picture of the role of ecoclimatic factors in this process. Our comparative analyses show that almost all cooperatively breeding species evolved from family-living ancestors and that many of the ecoclimatic correlates that were previously thought to promote helping at the nest [3,6,37] may have favored instead the prerequisite transition toward family living. This finding highlights that helping at the nest is not the only social adaptation that can help birds deal with variable environmental conditions. For example, family living can reduce the mortality of independent juveniles [38] through parental protection against predators [39–41], easier offspring access to resources [42,43], increased offspring foraging efficiency [44], and a potential reduction of per capita investment in territoriality [45]. Furthermore, family living is associated with ample opportunities to socially acquire critical life skills [46] and potentially increase cognitive abilities [47]. These benefits of family life may improve offspring survival in productive but variable environments [38] and lead to higher grand-offspring fitness even in the absence of helping at the nest [48]. Notably, these direct fitness benefits accrue both during and outside of the breeding season and, most importantly, suggest that cooperation outside of the reproductive context facilitates the evolution of family living [18]. These insights allow us to reconsider the role of limited dispersal options (i.e., ecological constraints [3]) for the evolution of cooperative breeding. Both family living and cooperative breeding are associated with productive but variable habitats that may limit dispersal options; however, it is more likely that these conditions in fact facilitate family living by reducing the cost to parents [16] and offspring [18,26]. Thus, delayed dispersal is an adaptive life-history decision rather than a “best of a bad job” strategy reflecting dispersal constraints [49].

Earlier studies have reported a rather weak and variable influence of ecoclimatic factors on the distribution of cooperative breeding [9,23,50]. However, the effects of these predictors are likely to have been inadvertently misinterpreted by considering a single transition from non-family living to cooperative breeding. As shown above, the initial formation of family groups was likely to be associated with the occupancy of productive environments that facilitate family living [14,18]. In contrast, the subsequent evolution of cooperative breeding was likely to have been associated instead with a secondary occupancy of environments with more variable productivity. In years of low productivity, helping at the nest benefits both parents [23,25] and offspring [2,5,8], as these conditions increase the chance for parents to breed successfully and limit the chances of offspring to successfully breed independently, particularly in long-lived species [26]. In some short-lived cooperative breeders, mature offspring disperse nearby to breed independently, and proximity allows relatives to provide help at each other’s nests [8]. Low environmental productivity has also been suggested to favor cooperative breeding in mammals [51] and humans [52]. Moreover, a high within-group relatedness (i.e., family living) has been proposed to facilitate the evolution of eusociality in insects as well as cooperative breeding in mammals [21,53]. Therefore, a high enough but variable level of resources throughout the year favors the evolution of persistent kin groups and cooperation outside the reproductive context, while an additional increase in the variation in productivity may act as the condition favoring the subsequent evolution of cooperative breeding.

A recent comparative study suggested that high annual survival facilitates the evolution of cooperative breeding in birds [35]. Using these data but separating noncooperative breeders into non-family-living and family-living species shows that both cooperatively breeding and family-living species have a higher annual survival than non-family-living species (Phylogenetic Generalized Least Squares [PGLS] model: non-family living versus cooperative breeding: *p* = 0.00001, non-family living versus family living: *p* = 0.03; controlling for body size; *N* = 189 species). High annual survival allows prolonged parental investment into offspring [16,47] by providing offspring an incentive to remain with the parents beyond independence [38]. Moreover, it favors a delayed onset of independent reproduction [26], making cooperative breeding adaptive, particularly in variable environments where helpers at the nest can buffer reproductive failure in harsh years [6].

Our findings provide novel insights into the geographic distribution of different social systems, which has currently defied full explanation [9]. We speculate that the answer lies with an increase in the within year variance in environmental productivity. For example, several of the previously identified geographic hotspots of cooperative breeding (Southern Africa, Australia, Northern South America; [9]) underwent drastic climatic changes throughout the Eocene, from subtropical and tropical climates to seasonal savanna habitats or arid environments [54]. Accordingly, these environmental changes suggest these hotspot locations likely changed over time from favoring family living to favoring cooperative breeding.

In conclusion, our analyses reveal 2 key findings that provide a novel way of understanding the evolution of cooperation in birds and suggest a resolution for earlier equivocal findings [6,7,9,10,23]. First, family living enables coping with variable environmental conditions and increases offspring survival both within and outside the breeding season [38]. Subsequently, it sets the scene for the secondary evolution of cooperative breeding [5] when environments have become more variable throughout the year and during the breeding season. Second, we found that cooperative breeding among unrelated individuals is exceptional and likely has different evolutionary origins than family-based cooperative breeding (S2 Fig). Previous work suggested that this form of cooperative breeding arose through an alternative pathway, namely direct fitness benefits from reproductive sharing [13]. Overall, our analysis shows that considering path dependence is essential for understanding the evolution of complex adaptations, such as cooperative breeding, that may involve multiple independent evolutionary steps to be achieved [55].

## Materials and methods

We collected data on the social system, life history, and ecological parameters of bird species from the literature (see S1 Text). We used 3 different criteria to differentiate between the different social systems, using the known duration of family associations (i.e., the time offspring remain with parents beyond nutritional independence [14]), the occurrence of family groups during the non-breeding season (when the exact time offspring remain with parents beyond independence was unknown), or the occurrence of cooperative breeding [15] and the kin relationship of helpers [13] (see S1 Text). We did not categorize occasional cooperative breeding species as cooperative breeders [1] (based on the first 2 criteria above). Occasional cooperative breeding resembles interspecific feeding, in which individuals feed offspring of another species, and thus, different factors are likely to be associated with occasional cooperative breeding and regular cooperative breeding [1].

Species were categorized as sedentary (maximally engage in local movements) or migratory (short-, long-distance, and altitudinal migrants). Species that only use 1 food category were categorized as food specialists, whereas species that used at least 2 different food types were categorized as food generalists (see S1 Text for details on the food categorization). Habitat openness was calculated based on aerial images, following the International Union for Conservation of Nature (IUCN) Habitat Classification Scheme [56]. Nest type was categorized as a binary variable (cavity breeders: nests in cavities, cliffs, and caves; other nests: all other nest constructions). We used the mean body weight (combining male and female weight) and distinguished precocial from altricial species (categorizing semiprecocial species as precocial and semialtricial species as altricial).

Climatic variables were computed from data provided by the Climatic Research Unit Time Series 3.21 database at the University of East Anglia (http://catalogue.ceda.ac.uk/uuid/ac4ecbd554d0dd52a9b575d9666dc42d; downloaded 7 April 2014) and NASA (http://neo.sci.gsfc.nasa.gov/view.php?datasetId=MOD17A2_M_PSN; downloaded 5 December 2013). We calculated for each species:

- Precipitation: mean, within and between year variance, predictability during the whole year;
- Precipitation: mean, within and between year variance during the MGS only;
- Temperature: mean, within and between year variance, predictability during the whole year;
- Temperature: mean, within and between year variance during the MGS only;
- NPP: mean, variance, predictability during the whole year;
- NPP: mean, within and between year variance, predictability during the MGS only;
- MGS length;
- Habitat heterogeneity (according to [57]).

Since the variance often increases with the mean (i.e., Taylor’s law [58]), it has been suggested that the coefficient of variance may be a more appropriate measurement to assess climatic variability. Thus, we re-ran our analyses using the coefficient of variance where appropriate (for variables measured on absolute scales, i.e., precipitation, temperature). Both the PCA (S4 Table) and the multinomial model (S5 Table) resulted in qualitatively similar results as our main analyses, indicating that our choice of variability metric did not bias our results.

All statistical analyses were performed in R with the packages Diversitree [28], phytools [59], and MCMCglmm [29]. Ancestral state estimation was performed using the MuSSE function of the Diversitree package [28] on a consensus phylogeny estimated from a sample of 1,000 phylogenetic trees [27] with the maximum parsimony matrix method using the Hackett tree backbone. We note that using a consensus phylogeny with the Ericsson backbone returned qualitatively identical results. Also, using a model in which speciation and extinction rates were allowed to vary resulted qualitatively in the same results as the main models with diversification rates fixed to be equal across breeding modes (S6 Table). We fitted phylogenetically controlled multinomial models to our data using MCMCglmm [29]. The response variable in all models was a categorical representation of social system (3 nominal levels: non-family living, family living, and cooperative breeding). The phylogenetic random effect was modelled based on a recent phyla-wide phylogeny [27]. To account for the uncertainty of phylogeny estimation, we refitted the main model with 50 randomly selected trees from the posterior distribution of trees published in Jetz et al. [27]. Given that ancestral character reconstruction may be biased when characters influence diversification [60], we also used a phylogenetic controlled PCA (phyloPCA function in phytools) [59], resulting is a somewhat different PC structure (S7 Table). However, running our main model with this PC resulted qualitatively in the same results (S8 Table).

## Supporting information

S1 Fig A family group of Siberian jays. Siberian jays (*Perisoreus infaustus*) are an example of a family-living bird species where offspring remain with their parents but do not engage in helping at the nest. This social system is a pivotal steppingstone in the evolution of cooperative breeding, providing offspring with ample social learning opportunities to acquire life skills and prolonged parental investment. Thus, cooperation outside of the reproductive context facilitates the evolution of family living.
(TIF)

S2 Fig Estimated evolutionary transitions of bird social system including non-kin cooperatively breeding species. Transition model including all four social systems: non-family living species (No Fam), family living species (Fam), cooperatively breeding family living species (Coop), and non-kin cooperatively breeding species (NK-coop). The size of the circles proportional to the relative abundance of the four social systems.
(TIF)

S1 Table Taxonomic distribution (a) and geographic distribution (b) of the species included in our main analyses.
(DOCX)

S2 Table Principal component analyses of all climatic and continuous eco-climatic and life history parameters that potentially influence the occurrence of social system. Standardized loadings of the main contributors to each component are highlighted in bold. sqrt = square root transformed, ln = log transformed, var = variance, prcp = precipitation, MGS = mean growing season, NPP = net primary productivity, P = predictability.
(DOCX)

S3 Table Multinomial phylogenetically-controlled generalized linear mixed model comparing the effect of eco-climatic and life history variables on the evolution of social systems when longevity data are included in the model (N = 1023 species). Coefficients reflect the results of multinomial phylogenetic regression models with ‘cooperative families’ as the reference category. Significant factors are highlighted in bold. Analysis based on a consensus-tree [27], using the Hackett backbone [61].
(DOCX)

S4 Table Principal component analyses of all climatic and continuous eco-climatic and life history parameters that potentially influence the occurrence of social system, using coefficient of variance for rainfall and temperature. Standardized loadings of the main contributors to each component are highlighted in bold. sqrt = square root transformed, ln = log transformed, CV = coefficient of variance, var = variance, prcp = precipitation, MGS = mean growing season, NPP = net primary productivity, P = predictability, btw = between.
(DOCX)

S5 Table Multinomial phylogenetically controlled generalized linear mixed models comparing the effect of eco-climatic and life history variables on the evolution of non-family-living, family-living and cooperative breeding species; N = 2968 bird species (excluding cooperative breeding species with non-kin helpers only), using coefficient of variance for precipitation and temperature. Coefficients reflect the results of multinomial phylogenetic regression models with ‘cooperative families’ as the reference category in the analyses and thus not shown per se. Significant factors are highlighted in bold. The Principal Component Analyses resulting in PC1-8 is shown in S4 Table. The factor social system assessment specified whether it was assessed based on the time offspring remained with their parents beyond independence (using 50 days as a threshold to differentiate between non-family living and family living species), breeding behavior, or social information.
(DOCX)

S6 Table Transition rates between the three major social systems in MuSSE models with fixed and variable diversification (div) and extinction (ext) rates. nf: non-family living, fam: family living, coop: cooperative breeding.
(DOCX)

S7 Table Principal component analyses of all climatic and continuous eco-climatic and life history parameters that potentially influence the occurrence of social system, using a phylogenetic controlled PCA [62]. Standardized loadings of the main contributors to each component are highlighted in bold. sqrt = square root transformed, ln = log transformed, var = variance, prcp = precipitation, MGS = mean growing season, NPP = net primary productivity, P = predictability.
(DOCX)

S8 Table Multinomial phylogenetically-controlled generalized linear mixed model comparing the effect of eco-climatic and life history variables (based on a phylogenetic PC)on the evolution of social systems based on a consensus-tree [27] with the Hackett backbone [61]. Coefficients reflect the results of multinomial phylogenetic regression models with ‘cooperative families’ as the reference category. Significant factors are highlighted in bold. The results are quantitatively corresponding to the model including 50 trees and a normal PC (Table 1).
(DOCX)

S1 Text Detailed materials and methods. Detailed Materials and Methods. (DOCX)

S1 Data Data used for analyses. (XLSX)

## Abbreviations

MCMC: Markov chain Monte Carlo
MGS: mean growing season
NPP: net primary productivity
PC: principal component
PCA: principal component analysis
PGLS: Phylogenetic Generalized Least Squares
sqrt: square root transformed
