# Supplementary figures and images for: Family living sets the stage for cooperative breeding and ecological resilience in birds
Source: PLoS Biol. 2017 Jun 21;15(6):e2000483. doi: 10.1371/journal.pbio.2000483 (PMC5479502; doi:10.1371/journal.pbio.2000483)

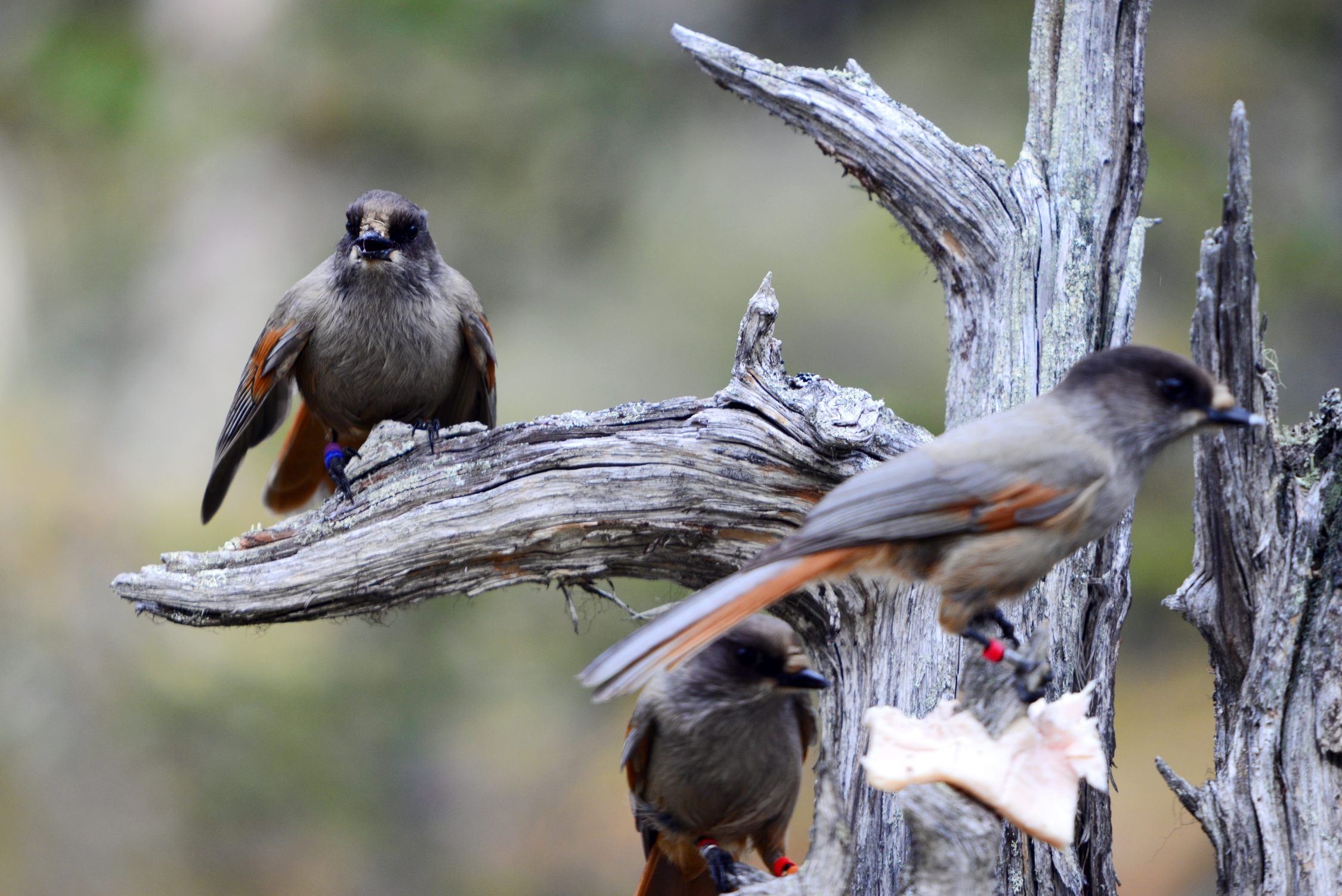

Supplement: S1 Fig — Siberian jays (Perisoreus infaustus) are an example of a family-living bird species where offspring remain with their parents but do not engage in helping at the nest. This social system is a pivotal steppingstone in the evolution of cooperative breeding, providing offspring with ample social learning opportunities to acquire life skills and prolonged parental investment. Thus, cooperation outside of the reproductive context facilitates the evolution of family living. (TIF) [file pbio.2000483.s001.tif]

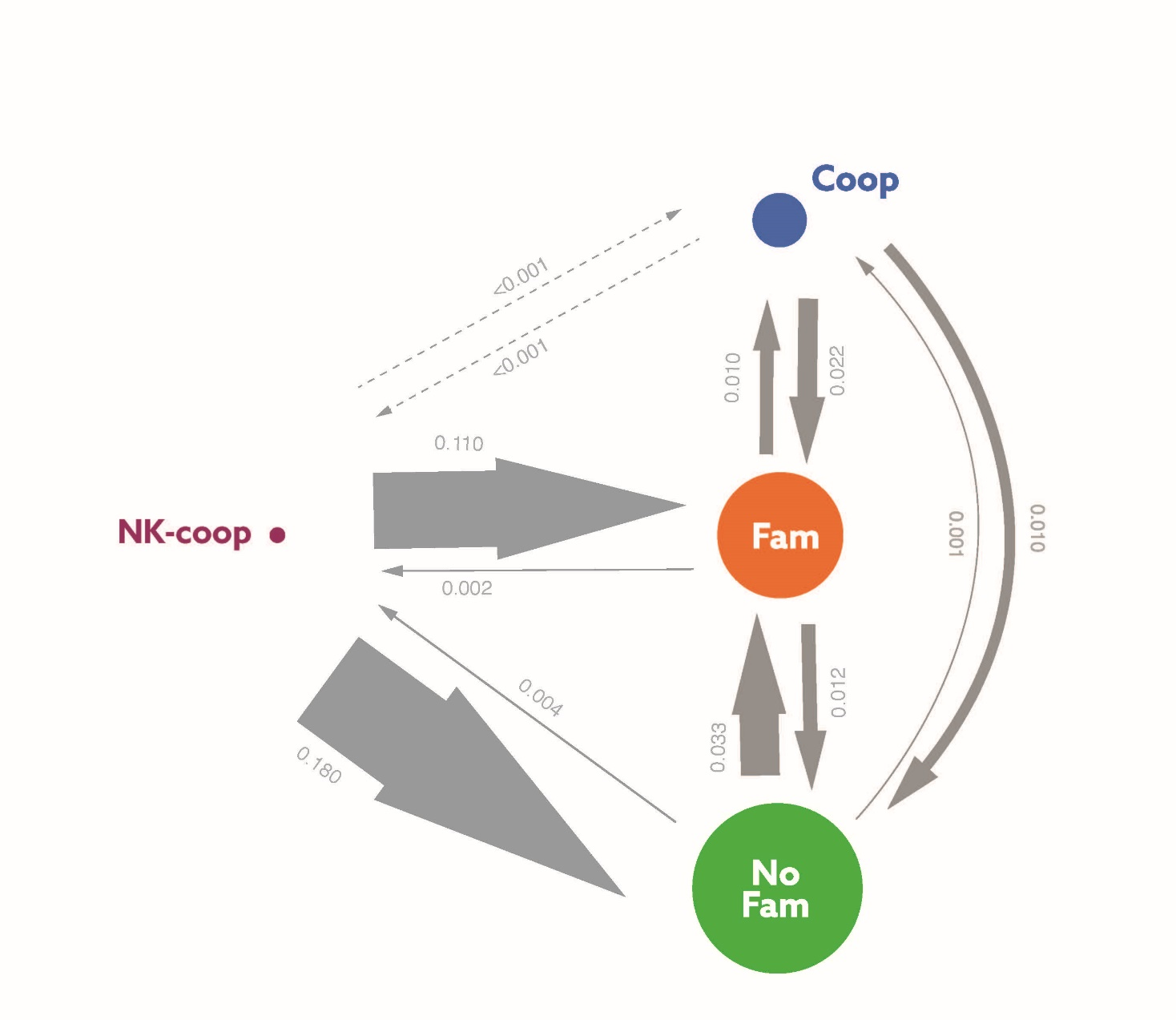

Supplement: S2 Fig — Transition model including all four social systems: non-family living species (No Fam), family living species (Fam), cooperatively breeding family living species (Coop), and non-kin cooperatively breeding species (NK-coop). The size of the circles proportional to the relative abundance of the four social systems. (TIF) [file pbio.2000483.s002.tif]
