# Supplementary material for: Family living sets the stage for cooperative breeding and ecological resilience in birds
Source: PLoS Biol. 2017 Jun 21;15(6):e2000483. doi: 10.1371/journal.pbio.2000483 (PMC5479502; doi:10.1371/journal.pbio.2000483)
Supplement: S1 Table — Taxonomic distribution (a) and geographic distribution (b) of the species included in our main analyses. (DOCX) [file pbio.2000483.s003.docx]

**S1 Table**

a)

| Order | non-family living | family living | cooperative family | sum |
| --- | --- | --- | --- | --- |
| ACCIPITRIFORMES | 47 | 35 | 2 | 84 |
| ANSERIFORMES | 47 | 30 | 0 | 77 |
| APODIFORMES | 273 | 0 | 4 | 277 |
| APTERYGIFORMES | 2 | 0 | 1 | 3 |
| BUCEROTIFORMES | 7 | 9 | 15 | 31 |
| CAPRIMULGIFORMES | 9 | 6 | 0 | 15 |
| CASUARIIFORMES | 2 | 2 | 0 | 4 |
| CHARADRIIFORMES | 78 | 38 | 0 | 116 |
| CICONIIFORMES | 6 | 2 | 0 | 8 |
| COLIIFORMES | 0 | 0 | 2 | 2 |
| COLUMBIFORMES | 95 | 2 | 0 | 97 |
| CORACIIFORMES | 14 | 2 | 20 | 36 |
| CUCULIFORMES | 59 | 1 | 0 | 60 |
| EURYPYGIFORMES | 1 | 0 | 0 | 1 |
| FALCONIFORMES | 10 | 4 | 1 | 15 |
| GALLIFORMES | 8 | 75 | 1 | 84 |
| GAVIIFORMES | 5 | 0 | 0 | 5 |
| GRUIFORMES | 14 | 14 | 12 | 40 |
| MESITORNITHIFORMES | 1 | 1 | 1 | 3 |
| MUSOPHAGIFORMES | 0 | 1 | 3 | 4 |
| OPISTHOCOMIFORMES | 0 | 0 | 1 | 1 |
| OTIDIFORMES | 5 | 10 | 0 | 15 |
| PASSERIFORMES | 819 | 581 | 280 | 1680 |
| PELECANIFORMES | 45 | 2 | 1 | 48 |
| PHOENICOPTERIFORMES | 5 | 0 | 0 | 5 |
| PICIFORMES | 37 | 53 | 33 | 123 |
| PODICIPEDIFORMES | 11 | 4 | 0 | 15 |
| PSITTACIFORMES | 16 | 39 | 5 | 60 |
| PTEROCLIDIFORMES | 1 | 0 | 0 | 1 |
| RHEIFORMES | 0 | 1 | 0 | 1 |
| STRIGIFORMES | 30 | 13 | 1 | 44 |
| STRUTHIONIFORMES | 0 | 1 | 0 | 1 |
| SULIFORMES | 9 | 0 | 0 | 9 |
| TINAMIFORMES | 1 | 0 | 0 | 1 |
| TROGONIFORMES | 0 | 1 | 0 | 1 |
| sum | 1657 | 927 | 383 | 2967 |

b)

| region | non-family living | family living | cooperative family | sum |
| --- | --- | --- | --- | --- |
| Africa | 325 | 257 | 148 | 730 |
| Antarctic | 1 | 1 | 0 | 2 |
| Australia | 157 | 125 | 96 | 378 |
| Holarctic | 53 | 12 | 0 | 65 |
| Indomalayan | 114 | 66 | 26 | 206 |
| Nearctic | 217 | 39 | 13 | 268 |
| Neotropical | 555 | 338 | 83 | 976 |
| Palearctic | 168 | 67 | 11 | 246 |
| Widespread | 67 | 22 | 6 | 95 |
| sum | 1657 | 927 | 383 | 2967 |
