# Supplementary material for: Family living sets the stage for cooperative breeding and ecological resilience in birds
Source: PLoS Biol. 2017 Jun 21;15(6):e2000483. doi: 10.1371/journal.pbio.2000483 (PMC5479502; doi:10.1371/journal.pbio.2000483)
Supplement: S2 Table — Standardized loadings of the main contributors to each component are highlighted in bold. sqrt = square root transformed, ln = log transformed, var = variance, prcp = precipitation, MGS = mean growing season, NPP = net primary productivity, P = predictability. (DOCX) [file pbio.2000483.s004.docx]

**Table S2.**

| eco-climatic variable | Variable rainfall among years (PC1) | Mean growing season duration (PC2) | Within year variance in productivity (PC3) | Precipitation predictability (PC4) | Among year variance in MGS's NPP (PC5) | Residual geographic range (PC6) | Residual habitat openness (PC7) | Residual body size (PC8) | Unique-ness |
| --- | --- | --- | --- | --- | --- | --- | --- | --- | --- |
|  |  |  |  |  |  |  |  |  |  |
|  |  |  |  |  |  |  |  |  |  |
| sqrt (var prcp MGS) | **0.94** | 0.2 | 0.07 | 0.01 | 0.05 | -0.11 | -0.07 | -0.02 | 0.06 |
| sqrt (prcp var) | **0.93** | 0.22 | 0.09 | 0 | 0.06 | -0.1 | -0.08 | -0.03 | 0.06 |
| LN (prcp inter year var MGS) | **0.85** | 0.33 | 0.09 | 0.17 | -0.04 | -0.11 | -0.15 | -0.03 | 0.09 |
| sqrt (prcp mean MGS) | **0.77** | 0.3 | 0.27 | 0.39 | 0.06 | -0.15 | -0.14 | 0 | 0.04 |
| sqrt (prcp mean) | **0.76** | 0.35 | 0.29 | 0.36 | 0.05 | -0.15 | -0.15 | -0.01 | 0.05 |
| temp mean MGS | **0.63** | 0.45 | -0.3 | 0.05 | 0.46 | -0.07 | -0.05 | -0.03 | 0.09 |
| temp mean | **0.58** | **0.54** | -0.31 | -0.03 | 0.42 | -0.07 | -0.05 | -0.05 | 0.09 |
| NPP P | **-0.57** | -0.46 | -0.35 | -0.25 | -0.4 | 0.13 | 0.18 | 0.04 | 0.07 |
| NPP mean | **0.53** | **0.53** | 0.46 | 0.22 | -0.03 | -0.23 | -0.2 | -0.03 | 0.08 |
| NPP mean MGS | **0.52** | 0.46 | **0.51** | 0.25 | -0.04 | -0.23 | -0.2 | -0.03 | 0.1 |
| MGS duration | 0.33 | **0.84** | 0.08 | -0.17 | 0.03 | -0.07 | -0.11 | -0.09 | 0.12 |
| LN (temp inter year var MGS) | -0.47 | **-0.71** | 0.2 | -0.12 | -0.24 | 0.18 | 0.07 | 0.01 | 0.13 |
| LN (temp var) | -0.45 | **-0.66** | 0.09 | -0.38 | -0.22 | 0.26 | 0.13 | 0.09 | 0.06 |
| temp P | 0.46 | **0.65** | -0.18 | 0.37 | 0.26 | -0.23 | -0.07 | -0.02 | 0.07 |
| LN (temp var MGS) | -0.44 | **-0.62** | 0.09 | -0.44 | -0.21 | 0.27 | 0.13 | 0.09 | 0.07 |
| LN (NPP var) | 0.12 | -0.06 | **0.95** | 0.1 | 0.1 | 0.03 | -0.07 | -0.01 | 0.05 |
| sqrt (NPP var MGS) | 0.06 | -0.11 | **0.95** | 0.1 | 0.05 | 0.01 | -0.06 | -0.01 | 0.06 |
| prcp P | 0.18 | 0.04 | 0.34 | **0.85** | 0.18 | -0.15 | -0.03 | 0.01 | 0.07 |
| LN (NPP inter year var MGS) | 0.03 | 0.19 | 0.21 | 0.17 | **0.86** | -0.01 | -0.02 | 0 | 0.15 |
| habitat heterogeneity | -0.26 | -0.11 | -0.01 | -0.24 | -0.24 | **0.84** | 0.08 | 0.05 | 0.09 |
| LN (breeding range area) | -0.1 | -0.42 | 0.01 | 0.02 | 0.4 | **0.7** | 0.11 | 0.05 | 0.14 |
| habitat openness | -0.26 | -0.19 | -0.16 | -0.06 | -0.04 | 0.13 | **0.91** | 0.1 | 0.01 |
| LN (body weight) | -0.03 | -0.09 | -0.02 | -0.01 | 0 | 0.06 | 0.08 | **0.99** | 0 |
|  |  |  |  |  |  |  |  |  |  |
| SS loadings | 6.38 | 4.35 | 3.06 | 1.85 | 1.79 | 1.69 | 1.12 | 1.03 |  |
| % Cumulative Variance explained | 0.28 | 0.47 | 0.60 | 0.68 | 0.76 | 0.83 | 0.88 | 0.92 |  |
