# Supplementary material for: Family living sets the stage for cooperative breeding and ecological resilience in birds
Source: PLoS Biol. 2017 Jun 21;15(6):e2000483. doi: 10.1371/journal.pbio.2000483 (PMC5479502; doi:10.1371/journal.pbio.2000483)
Supplement: S3 Table — Coefficients reflect the results of multinomial phylogenetic regression models with ‘cooperative families’ as the reference category. Significant factors are highlighted in bold. Analysis based on a consensus-tree [27], using the Hackett backbone [61]. (DOCX) [file pbio.2000483.s005.docx]

**Table S3.**

|  | Family living species (reference) vs non-family living species: | | |  | Family living species (reference) vs cooperative breeding species: | | |
| --- | --- | --- | --- | --- | --- | --- | --- |
| Factor | posterior mean | 95% CI (lower; upper) | pMCMC |  | posterior mean | 95% CI (lower; upper) | pMCMC |
| Intercept | 0.06 | -1.42 ; 1.31 | 0.90 |  | -0.29 | -1.52 ; 0.98 | 0.64 |
| Variance in rainfall (PC1) | **0.81** | **0.30 ; 1.36** | **0.002** |  | -0.12 | -0.73 ; 0.47 | 0.74 |
| Mean growing season duration (PC2) | **-0.68** | **-1.13 ; -0.26** | **<0.001** |  | 0.11 | -0.43 ; 0.72 | 0.70 |
| Within year variance in productivity (PC3) | 0.31 | -0.04 ; 0.62 | 0.056 |  | -0.33 | -0.76 ; 0.17 | 0.18 |
| Precipitation predictability) (PC4) | 0.12 | -0.29 ; 0.61 | 0.60 |  | -0.06 | -0.74 ; 0.58 | 0.87 |
| Among year variance in MGS's NPP (PC5) | -0.34 | -0.82 ; 0.17 | 0.19 |  | 0.54 | -0.05 ; 1.23 | 0.10 |
| Residual geographic range (PC6) | 0.14 | -0.16 ; 0.53 | 0.41 |  | -0.24 | -0.69 ; 0.25 | 0.31 |
| Residual habitat openness (PC7) | **0.49** | **0.07 ; 0.86** | **0.009** |  | -0.30 | -0.83 ; 0.20 | 0.27 |
| Residual body size (PC8) | **-1.11** | **-1.79 ; -0.48** | **<0.001** |  | -0.40 | -1.09 ; 0.31 | 0.29 |
| Chick development modus (altricial vs precocial)^‡^ | -0.11 | -1.36 ; 1.27 | 0.86 |  | -0.30 | -1.47 ; 1.00 | 0.65 |
| Food specialization (generalist vs specialist) ^‡^ | -0.74 | -1.49 ; -0.02 | 0.052 |  | 0.64 | -0.16 ; 1.48 | 0.15 |
| Sedentariness (sedentary vs migratory) ^‡^ | -0.46 | -1.28 ; 0.26 | 0.25 |  | 0.98 | -0.03 ; 2.08 | 0.06 |
| Nest type (cavity vs open nesting) ^‡^ | -0.30 | -1.27 ; 0.61 | 0.56 |  | -0.78 | -1.80 ; 0.21 | 0.12 |
| Longevity | -0.02 | -0.08 ; 0.04 | 0.53 |  | -0.01 | -0.09 ; 0.05 | 0.74 |
| Sampling effort (number of Zoological Record hits) | **0** | **0 ; 0** | **0.034** |  | 0 | 0 ; 0 | 0.40 |
| Social system assessment –breeding | -0.48 | -1.56 ; 0.67 | 0.39 |  | 2.41 | 1.28 ; 3.33 | <0.001 |
| Social system assessment –social | 0.33 | -0.41 ; 1.00 | 0.37 |  | -2.47 | -3.55 ; -1.54 | <0.001 |

^‡^ Reference level is the first category in these lists
