# Supplementary material for: Family living sets the stage for cooperative breeding and ecological resilience in birds
Source: PLoS Biol. 2017 Jun 21;15(6):e2000483. doi: 10.1371/journal.pbio.2000483 (PMC5479502; doi:10.1371/journal.pbio.2000483)
Supplement: S4 Table — Standardized loadings of the main contributors to each component are highlighted in bold. sqrt = square root transformed, ln = log transformed, CV = coefficient of variance, var = variance, prcp = precipitation, MGS = mean growing season, NPP = net primary productivity, P = predictability, btw = between. (DOCX) [file pbio.2000483.s006.docx]

**Table S4.**

| eco-climatic variable | Harshness (PC1) | Rainfall variability (PC2) | Within year variance in productivity (PC3) | Residual geographic range (PC4) | Precipitation unpredicta-bility (PC5) | Among year variance in MGS's NPP (PC6) | Residual habitat openness (PC7) | Residual body weight (PC8) | Unique-ness |
| --- | --- | --- | --- | --- | --- | --- | --- | --- | --- |
| SQRT.Prcp.Mean | **-0.71** | **-0.55** | 0.26 | -0.06 | -0.13 | -0.14 | -0.15 | -0.03 | 0.05 |
| SQRT.CV.Prcp.Mean | 0 | **0.94** | -0.23 | 0.11 | -0.02 | -0.05 | 0.1 | 0.02 | 0.04 |
| Prcp.P | -0.2 | **-0.52** | 0.27 | 0.01 | **-0.67** | 0.15 | 0.08 | -0.04 | 0.13 |
| Temp.Mean | **-0.93** | 0.14 | -0.1 | 0.04 | 0.04 | 0.06 | -0.12 | -0.03 | 0.08 |
| SQRT.CV.Temp.Mean | **0.92** | 0.11 | 0.05 | 0.22 | 0.06 | -0.1 | 0.06 | 0.1 | 0.06 |
| Temp.P | **-0.9** | -0.1 | -0.09 | -0.2 | -0.15 | 0.15 | -0.01 | -0.03 | 0.08 |
| NPP.Mean | **-0.64** | -0.45 | 0.43 | -0.29 | 0.04 | -0.12 | -0.18 | -0.05 | 0.07 |
| LN.NPP.Var | 0.12 | -0.41 | **0.88** | 0.06 | -0.05 | 0.1 | -0.07 | -0.03 | 0.03 |
| NPP.P | **0.77** | 0.35 | -0.35 | 0.03 | 0.1 | -0.21 | 0.18 | 0.06 | 0.07 |
| SQRT.Prcp.Mean.MGS | **-0.71** | **-0.54** | 0.25 | -0.04 | -0.19 | -0.14 | -0.14 | -0.02 | 0.06 |
| SQRT.CV.Prcp_within_MGS.Mean | -0.07 | **0.94** | -0.2 | 0.06 | 0.11 | -0.06 | 0.08 | 0 | 0.04 |
| SQRT.CV.Prcp_btw_MGS.Mean | 0.28 | **0.73** | -0.36 | -0.02 | 0.17 | -0.21 | 0.05 | 0.05 | 0.19 |
| Temp.Mean.MGS | **-0.93** | 0.1 | -0.11 | 0.09 | -0.08 | 0.06 | -0.12 | 0 | 0.08 |
| LN.CV.Temp_within_MGS.Mean | **0.91** | 0.07 | 0.09 | 0.22 | 0.11 | -0.1 | 0.04 | 0.08 | 0.08 |
| SQRT.CV.Temp_btw_MGS.Mean | **0.91** | 0.07 | 0.08 | 0.17 | 0.01 | -0.16 | 0.03 | 0.01 | 0.12 |
| NPP.Mean.MGS | **-0.57** | -0.49 | 0.48 | -0.26 | -0.01 | -0.13 | -0.19 | -0.04 | 0.09 |
| SQRT.NPP.Var.MGS | 0.2 | -0.4 | **0.86** | 0.03 | -0.07 | 0.07 | -0.07 | -0.02 | 0.04 |
| LN.NPP.InterYearVar.MGS | -0.37 | -0.16 | 0.13 | 0.15 | -0.11 | **0.85** | -0.03 | 0.01 | 0.06 |
| HabitatHeterogeneity | 0.33 | 0.12 | -0.01 | **0.62** | 0.53 | -0.16 | 0.15 | 0.02 | 0.16 |
| LN.BreedingRangeArea | 0.23 | 0.08 | 0.03 | **0.88** | -0.08 | 0.19 | 0.05 | 0.11 | 0.11 |
| MGS.duration | **-0.73** | -0.05 | 0.13 | -0.28 | 0.46 | 0.05 | -0.06 | -0.13 | 0.13 |
| habitatopenness | 0.29 | 0.21 | -0.14 | 0.11 | -0.01 | -0.03 | **0.88** | 0.16 | 0.04 |
| LN.BodyWeight | 0.12 | 0.03 | -0.04 | 0.1 | 0.01 | 0.01 | 0.13 | **0.98** | 0.006 |
