# Supplementary material for: Family living sets the stage for cooperative breeding and ecological resilience in birds
Source: PLoS Biol. 2017 Jun 21;15(6):e2000483. doi: 10.1371/journal.pbio.2000483 (PMC5479502; doi:10.1371/journal.pbio.2000483)
Supplement: S5 Table — Coefficients reflect the results of multinomial phylogenetic regression models with ‘cooperative families’ as the reference category in the analyses and thus not shown per se. Significant factors are highlighted in bold. The Principal Component Analyses resulting in PC1-8 is shown in S4 Table. The factor social system assessment specified whether it was assessed based on the time offspring remained with their parents beyond independence (using 50 days as a threshold to differentiate between non-family living and family living species), breeding behavior, or social information. (DOCX) [file pbio.2000483.s007.docx]

**Table S5.**

|  | Family living species (reference) vs non-family living species: | | | Family living species (reference) vs cooperative breeding species: | | | |
| --- | --- | --- | --- | --- | --- | --- | --- |
| Factor | Mean estimated effect^†^ | 95% CI (lower; upper) | *p MCMC* | Mean estimated effect^†^ | 95% CI (lower; upper) | *p MCMC* |  |
| Intercept | -0.94 | -2.23 ; 0.27 | 0.13 | 0.17 | -1.03 ; 1.55 | 0.79 |  |
| Variable rainfall (PC1) | -0.2 | -0.15 ; 0.54 | 0.24 | 0.81 | **0.55 ; 1.08** | **<0.001** |  |
| Mean growing season duration (PC2) | 0.21 | -0.06 ; 0.5 | 0.14 | -0.11 | -0.3 ; 0.07 | 0.28 |  |
| Within year variance in productivity (PC3) | **-0.33** | **-0.6 ; -0.06** | **0.011** | 0.16 | -0.03 ; 0.34 | 0.09 |  |
| Precipitation predictability (PC4) | 0 | -0.26 ; 0.27 | 0.96 | 0.12 | -0.06 ; 0.29 | 0.22 |  |
| Among year variance in MGS's NPP (PC5) | -0.1 | -0.34 ; 0.17 | 0.45 | -0.06 | -0.26 ; 0.14 | 0.55 |  |
| Residual geographic range (PC6) | 0.15 | -0.08 ; 0.38 | 0.21 | **-0.23** | **-0.43 ; -0.04** | **0.015** |  |
| Residual habitat openness (PC7) | -0.16 | -0.48 ; 0.11 | 0.27 | 0.18 | -0.02 ; 0.41 | 0.1 |  |
| Residual body size (PC8) | -0.08 | -0.53 ; 0.36 | 0.72 | **-0.93** | **-1.42 ; -0.49** | **<0.001** |  |
| Chick development modus (altrical vs precocial)^‡^ | -0.89 | -2.01 ; 0.25 | 0.13 | -0.48 | -1.77 ; 0.6 | 0.42 |  |
| Food specialization (generalist vs specialist) ^‡^ | 0.52 | -0.06 ; 1.01 | 0.06 | **-0.56** | **-1.02 ; -0.13** | **0.019** |  |
| Sedentariness (sedentary vs migratory) ^‡^ | 0.58 | -0.3 ; 1.41 | 0.19 | **-0.91** | **-1.44 ; -0.31** | **<0.001** |  |
| Nest type (cavity vs open nesting) ^‡^ | -0.29 | -1.12 ; 0.47 | 0.46 | -0.35 | -0.97 ; 0.25 | 0.31 |  |
| Social system assessment –breeding | **4.33** | **3.62 ; 5.08** | **<0.001** | **-1.06** | **-1.95 ; -0.12** | **0.026** |  |
| Social system assessment –social | **-3.76** | **-4.38 ; -3.14** | **<0.001** | **-0.02** | -0.42 ; 0.4 | 0.95 |  |

^‡^ Reference level is the first category in these lists
