# Supplementary material for: Family living sets the stage for cooperative breeding and ecological resilience in birds
Source: PLoS Biol. 2017 Jun 21;15(6):e2000483. doi: 10.1371/journal.pbio.2000483 (PMC5479502; doi:10.1371/journal.pbio.2000483)
Supplement: S6 Table — nf: non-family living, fam: family living, coop: cooperative breeding. (DOCX) [file pbio.2000483.s008.docx]

**Table S6.**

|  | nf-fam | nf-coop | fam-nf | fam-coop | coop-nf | coop-fam |
| --- | --- | --- | --- | --- | --- | --- |
| fixed div/ext rates | 0.015 | 0.0020 | 0.034 | 0.011 | 0.013 | 0.02 |
|  |  |  |  |  |  |  |
| variable div/ext rates | 0.010 | 0.0017 | 0.040 | 0.120 | 0.009 | 0.02 |
