# Supplementary material for: Family living sets the stage for cooperative breeding and ecological resilience in birds
Source: PLoS Biol. 2017 Jun 21;15(6):e2000483. doi: 10.1371/journal.pbio.2000483 (PMC5479502; doi:10.1371/journal.pbio.2000483)
Supplement: S7 Table — Standardized loadings of the main contributors to each component are highlighted in bold. sqrt = square root transformed, ln = log transformed, var = variance, prcp = precipitation, MGS = mean growing season, NPP = net primary productivity, P = predictability. (DOCX) [file pbio.2000483.s009.docx]

**Table S7.**

| eco-climatic variable | Harshness PC1 | NPP stability PC2 | Precipitation stability PC3 | Residual geogr. range (PC4) | Growing season duration PC5 | Residual body size (PC6) | Residual habitat openness (PC7) |
| --- | --- | --- | --- | --- | --- | --- | --- |
| sqrt (prcp mean) | **-0.87** | -0.29 | -0.26 | 0.02 | -0.11 | -0.01 | 0.01 |
| sqrt (prcp var) | **-0.79** | 0.04 | **-0.53** | -0.08 | -0.05 | 0.00 | 0.02 |
| prcp P | -0.33 | **-0.65** | 0.24 | -0.08 | -0.49 | 0.03 | -0.08 |
| temp mean | **-0.69** | 0.52 | 0.09 | -0.28 | 0.19 | 0.00 | 0.04 |
| LN (temp var) | **0.88** | -0.23 | -0.24 | -0.01 | 0.14 | 0.02 | 0.03 |
| temp P | **-0.85** | 0.28 | 0.24 | 0.01 | -0.15 | -0.04 | -0.03 |
| NPP mean | **-0.74** | -0.39 | 0.11 | 0.28 | 0.31 | -0.01 | 0.10 |
| LN (NPP var) | -0.15 | **-0.92** | 0.03 | 0.04 | 0.14 | 0.00 | 0.09 |
| NPP P | **0.78** | 0.42 | -0.21 | 0.20 | -0.19 | 0.03 | -0.03 |
| sqrt (prcp mean MGS) | **-0.86** | -0.31 | -0.27 | -0.02 | -0.16 | -0.01 | 0.00 |
| sqrt (var prcp MGS) | **-0.78** | 0.04 | **-0.54** | -0.09 | -0.06 | 0.00 | 0.02 |
| LN (prcp inter year var MGS) | **-0.80** | 0.02 | -0.45 | 0.00 | -0.07 | -0.01 | -0.01 |
| temp mean MGS | **-0.72** | 0.45 | 0.06 | -0.35 | 0.10 | 0.01 | 0.00 |
| LN (temp var MGS) | **0.86** | -0.19 | -0.24 | 0.00 | 0.21 | 0.01 | 0.05 |
| LN (temp inter year var MGS) | **0.78** | -0.43 | -0.18 | 0.00 | -0.06 | 0.07 | -0.02 |
| NPP mean MGS | **-0.69** | **-0.50** | 0.09 | 0.27 | 0.28 | 0.00 | 0.09 |
| sqrt (NPP var MGS) | -0.10 | **-0.92** | 0.03 | 0.09 | 0.11 | 0.02 | 0.09 |
| LN (NPP inter year var MGS) | -0.35 | -0.32 | 0.46 | **-0.52** | 0.00 | -0.06 | -0.07 |
| habitat heterogeneity | 0.44 | -0.03 | -0.28 | -0.37 | 0.44 | -0.19 | 0.02 |
| LN (breeding range area) | 0.30 | -0.42 | -0.08 | **-0.70** | 0.10 | -0.10 | -0.09 |
| MGS duration | **-0.54** | 0.38 | 0.15 | 0.22 | **0.50** | -0.08 | 0.14 |
| habitat openness | 0.25 | 0.10 | 0.04 | -0.14 | -0.27 | -0.24 | **0.88** |
| LN (body weight) | 0.05 | -0.04 | 0.00 | 0.19 | -0.08 | **-0.94** | -0.23 |
|  |  |  |  |  |  |  |  |
| SS loadings | 3.11 | 2.03 | 1.26 | 1.19 | 1.09 | 1 | 0.95 |
|  |  |  |  |  |  |  |  |
| corresponding PC of main PCA (Table S2): | PC 1 & 2 | PC 3 & 4 | included in PC 1 | PC 5 & 6 | included in PC 2 | PC 8 | PC 7 |
