# Supplementary material for: Family living sets the stage for cooperative breeding and ecological resilience in birds
Source: PLoS Biol. 2017 Jun 21;15(6):e2000483. doi: 10.1371/journal.pbio.2000483 (PMC5479502; doi:10.1371/journal.pbio.2000483)
Supplement: S8 Table — Coefficients reflect the results of multinomial phylogenetic regression models with ‘cooperative families’ as the reference category. Significant factors are highlighted in bold. The results are quantitatively corresponding to the model including 50 trees and a normal PC (Table 1). (DOCX) [file pbio.2000483.s010.docx]

**Table S8.**

|  | Family living species (reference) vs non-family living species: | | |  | Family living species (reference) vs cooperative breeding species: | | |  |
| --- | --- | --- | --- | --- | --- | --- | --- | --- |
| factor | posterior mean | 95% CI (lower; upper) | pMCMC |  | posterior mean | 95% CI (lower; upper) | pMCMC | |
| Intercept | 0.18 | -1.11 ; 1.46 | 0.80 |  | -0.58 | -1.73 ; 0.68 | 0.36 | |
| Harshness (PC1) | **0.07** | **0.05 ; 0.1** | **< 0.001** |  | 0.00 | -0.03 ; 0.03 | 0.89 | |
| NPP stability (PC2) | **-0.08** | **-0.12 ; -0.04** | **< 0.001** |  | **0.08** | **0.02 ; 0.15** | **0.01** | |
| Precipitation stability (PC3) | -0.05 | -0.1 ; 0.01 | 0.058 |  | 0.03 | -0.04 ; 0.11 | 0.45 | |
| Residual geographic range (PC4) | 0.00 | -0.05 ; 0.06 | 0.89 |  | 0.05 | -0.03 ; 0.13 | 0.19 | |
| Growing season duration (PC5) | -0.05 | -0.12 ; 0.03 | 0.23 |  | -0.03 | -0.14 ; 0.08 | 0.55 | |
| Residual body size (PC6) | **-0.09** | **-0.13 ; -0.04** | **< 0.001** |  | -0.01 | -0.06 ; 0.04 | 0.71 | |
| Residual habitat openness (PC7) | **0.09** | **0.02 ; 0.15** | **0.011** |  | -0.09 | -0.2 ; 0 | 0.058 | |
| Chick development modus (altrical vs precocial)^‡^ | -0.68 | -1.8 ; 0.54 | 0.30 |  | -0.70 | -1.85 ; 0.48 | 0.23 | |
| Food specialization (generalist vs specialist) ^‡^ | **-0.57** | **-0.98 ; -0.18** | **0.006** |  | 0.52 | -0.01 ; 1.15 | 0.069 | |
| Sedentariness (sedentary vs migratory) ^‡^ | **-0.88** | **-1.46 ; -0.3** | **0.002** |  | 0.47 | -0.32 ; 1.28 | 0.25 | |
| Nest type (cavity vs open nesting) ^‡^ | -0.36 | -1 ; 0.26 | 0.30 |  | -0.32 | -1.1 ; 0.37 | 0.40 | |
| Social system assessment –breeding | **-1.15** | **-2 ; -0.2** | **0.002** |  | **4.35** | **3.65 ; 5.16** | **< 0.001** | |
| Social system assessment –social | -0.04 | -0.46 ; 0.36 | 0.83 |  | **-3.75** | **-4.37 ; -3.05** | **< 0.001** | |

^‡^ Reference level is the first category in these lists

**References**

1. Jetz W, Thomas GH, Joy JB, Hartmann K, Mooers AO. The global diversity of birds in space and time. Nature. 2012;491(7424):444-8. doi: 10.1038/nature11631.

2. Hackett SJ, Kimball RT, Reddy S, Bowie RCK, Braun EL, Braun MJ, et al. A Phylogenomic Study of Birds Reveals Their Evolutionary History. Science. 2008;320(5884):1763-8. doi: 10.1126/science.1157704.
