# Supplementary material for: Family living sets the stage for cooperative breeding and ecological resilience in birds
Source: PLoS Biol. 2017 Jun 21;15(6):e2000483. doi: 10.1371/journal.pbio.2000483 (PMC5479502; doi:10.1371/journal.pbio.2000483)
Supplement: S1 Text — (DOCX) [file pbio.2000483.s011.docx]

**Detailed Materials and Methods:**

**Categorization of social system**

We categorized the species into non-family living, family living and cooperative breeding families based on published datasets [1-3] and data extracted from broad species accounts (Birds of the Western Palearctic [4], Handbook of the Birds of the World [5], Handbook of Australian, New Zealand & Antarctic Birds [6], Roberts Birds of Southern Africa [7], Birds of North America online [8], Handbook of the Birds of India and Pakistan [9]), and lineage specific handbooks [10-30].

We used three different criteria to differentiate between the social systems. For all species where the time offspring stay beyond nutritional independence with their parents is known (N=1053 species), we used a quantitative threshold of 50 days post-independence to differentiate non-family living (<50d) from family living species (≥50d). The logic behind this threshold is that species with a post-independence period of less than 50d show a marked higher body-mass scaled annual reproductive investment (details given in [31]. Observe that we did not use the body-mass scaled annual reproductive investment in our analyses but only as an objective tool to differentiate between non-family and family living species. This threshold indicates a life-history trade-off between current reproductive investment and prolonged offspring investment (i.e. family living), and falls together with seasonal environmental variation where offspring in family living species postpone dispersal beyond the onset of less favorable autumn conditions [31]. This information was combined with the information on whether a species breeds cooperatively or not [1,3] (see below).

For species where the time offspring stay beyond nutritional independence was unknown, we used information of their breeding systems or social information to categorize them into the different social systems. We categorized species as non-family living if social information specifically confirmed that offspring leave parents soon after independence, or parents do not interact with offspring once they fledged (N=1022 species). For N=23 species the maximum time offspring remain associated with their parents was not specified, but it was apparent that offspring stayed well beyond 50 days after independence with their parents (e.g. family groups are seen throughout the year or well beyond the end of the breeding season). Accordingly, these species were categorized as family living. Species were categorized as cooperative breeding families if cooperation was kin based [3] also when the exact time offspring remain with parents beyond independence was unknown (N=221 species). We controlled in our models for the potentially confounding effects of having classified the social system using three different sources of information (labelled social system assessment).

In contrast to earlier studies [1,32-35], we differentiated between species where cooperation occurred within families and species where cooperation occurred among unrelated individuals (i.e., non-kin cooperatively breeding species) [3]. In a majority of the latter species, more than two adults are involved in reproduction [3] and thus, the incentive to cooperate are direct fitness benefits. Moreover, the species that Cockburn [36] identified as occasional cooperative breeder were not classified as cooperative breeders, but categorized as non-family or family living species [37]. For example, helpers at the nest have been observed in several blue tit *Parus caeruleus* or house sparrow *Passer domesticus* populations, however, it always occurred at very low levels [36,37]. For species that express between population variation in their social system, for example carrion crow *Corvus corone [38]* or green jay *Cyanocorax yncas* [39], we assigned social system where offspring stay longest time with their parents beyond independence (non-family living < family living < cooperative breeding families).

**Categorization of migratory status, feeding habits and nesting type**

We used the same sources to extract information on ecological, social and life-history traits of species. We choose traits that earlier comparative studies identified to influence the occurrence of cooperative breeding or family living [32,40-46]. Species were categorized as sedentary if they only engage in local movements, whereas short- and long-distance as well as altitudinal migrants were categorized as migratory. Species that only use one of the food types listed below were categorized as food specialists whereas species that used at least two different food types were categorized as food generalists. We differentiated the following 17 food categories:

- beeswax
- bones
- carrion
- crustaceans
- eggs of vertebrates
- fish
- flowers
- fruits
- insects and invertebrates (including their eggs and larvae)
- mollusks
- nectar
- nuts
- plant materials (leaves and other green tissue)
- pollen
- sap
- seeds
- vertebrates.

Habitat openness was calculated based on the IUCN Habitat Classification Scheme [47] . We collected for all species their IUCN habitat categories [47] and ranked the different habitat types according to the importance for the species. For all habitat categories, we assessed amount of ground cover (excluding soft vegetation, i.e. grasses) following the method described in Mueller-Dombois [48], based on 10 randomly sampled location with the help of high-resolution satellite images covering the whole geographic range of the habitat category. We used the four principal habitat categories of each species to calculate the weighted average of the habitat openness (if 2 categories: 1^st^ category = 66.6%, 2^nd^ category = 33.3%; if 3 categories: 1^st^ category = 50%, 2^nd^ category = 30%, 3^rd^ category = 20%; if 4 categories: 1^st^ category = 50%, 2^nd^ category = 25%, 3^rd^ and 4^th^ category = 12.5%).

Nesting type was categorized as a binary variable based on nest type. Previous research suggested that cavity breeders are more likely to breed cooperatively, given that cavities can be a limited resource [33,43]. Thus, we categorized nests as cavities (nests in cavities, cliffs and caves), or non-cavities (all other nest constructions, i.e. closed or open and located in trees or shrubs, open nest on the ground).

**Body size, chick developmental mode and longevity data**

The body weight of species (in grams) was extracted from handbooks [4-30] or specific publications [49]. We used the mean body weight combining male and female weight. From the same sources we extracted the chick developmental mode distinguishing precocial from altricial species (categorizing semi-precocial species as precocial, and semi-altricial species as altricial [50]). We used the longevity data published in a recent paper [51], and complemented these data with longevity records of the Australian Bird and Bat Banding Scheme [52] and a review paper on parrots [53], following the methods outline in Valcu et al. [51]. If possible we used longevity records of non-captive birds, however, longevity records in captivity and the wild have been shown to be highly correlated [51]. Given that the research effort of a species influences the maximum observed longevity, the number of Zoological Record hits was included into these analyses (see [51] for more details).

**Eco-climatic parameters**

Precipitation and temperature data were obtained from the Climatic Research Unit Time Series 3.21 [54] (available at: <http://catalogue.ceda.ac.uk/uuid/ac4ecbd554d0dd52a9b575d9666dc42d>; Downloaded 07 Apr 14). Net primary production was obtained from MODIS [55] (available at:

<http://neo.sci.gsfc.nasa.gov/view.php?datasetId=MOD17A2_M_PSN>; Downloaded 05 Dec 2013). We extracted mean values, predictability (Colwell’s P [56]), and within- and between-year variances for each environmental variable first at a local scale (0.5 x 0.5 degree cells) and then averaged across the entire breeding distribution of a given species. Colwell’s P is an information theory based index that assesses the variation in the onset, duration and intensity of periodic phenomena, ranging from 0 (entirely unpredictable) to 1 (entirely predictable). Species distribution ranges were obtained from the 2010 IUCN Red List of Threatened Species v. 2010.4. (available at: [www.iucnredlist.org](http://www.iucnredlist.org). Downloaded 17 Apr 2012), and BirdLife International (available at: www.birdlife.org. Downloaded 17 Apr 2012). Because climatic unpredictability during the breeding season has been suggested to be of importance for evolution of cooperative breeding [57], we calculated eco-climatic variables both across the entire year and only within the period of year where there is significant local plant growth [58]. This proxy has been suggested to correlate with the duration of avian breeding seasons at a given locality. Accordingly, we calculated for each species:

- Precipitation: mean, within and between year variance, predictability during the whole year;
- Precipitation: mean, within and between year variance during the MGS only;
- Temperature: mean, within and between year variance, predictability during the whole year;
- Temperature: mean, within and between year variance during the MGS only;
- Net primary productivity (NPP): mean, variance, predictability during the whole year;
- NPP: mean, within and between year variance, predictability during the MGS only;
- Mean growing season length;
- Habitat heterogeneity (according to [59]).

**Statistical procedures**

We performed all statistical procedures in R version 3.1.0 [60].

**Ancestral state estimation**

Ancestral state estimation was performed using the MuSSE module of the *diversitree* package [61] on a consensus phylogeny estimated from a sample of 1000 phylogenetic trees [62] with the maximum parsimony matrix method. We performed this analysis based on the three social systems and assuming that all transitions between trait states were possible. MuSSE models the evolution of a categorical trait using a continuous-time Markov process [61], estimating relevant transition rates between pairs of trait states. In order to test hypotheses related to the pattern of transitions between social system states, we fitted a series of MuSSE models fixing relevant transition rates to zero or fixing them to be symmetrical (i.e. forward and backward transitions occur at equal rates). We defined all possible combinations of constraints of all six transition rates and ordered resulting models according to their AIC values [63]. The model with the lowest AIC value was considered as the best model explaining the evolution of social systems, and was used to generate the estimates of ancestral states across the tree using the stochastic mapping technique, as implemented in the Phytools package [64].

Given that the different social systems may influence the speciation and extinction rate, we also fitted a series of MuSSE models which speciation and extinction rates were allowed to vary across breeding modes. These models yielded the same qualitative results as the main models with diversification rates fixed to be equal across breeding modes (see table S6 for a comparison of transition rates in the two types of models).

**Principal component analysis**

Colinearity is a common issue in multivariate analysis, resulting from extensive correlation between predictors. We reduced the colinearity among our predictors by extracting a number principal components from the original set of continuous variables comprising species geographic range, body mass, habitat openness, habitat heterogeneity and all climatic parameters associated with precipitation, temperature and net primary productivity (see above). Before performing the PCA, we checked graphically the distributions of all variables, applied transformations when required to obtain more symmetrical distributions (see appendix Table 2), and centered and scaled our input variables. Principal components were extracted using the *principal* function (library *psych [65]*) with varimax rotation. In order to determine the number of PCs that suffices to summarize variation in the original predictors we simulated a distribution of eigenvalues of a matrix of uncorrelated random variates (of size equal to the number of predictors; function *parallel* in library *nFactors [66]*) and used it in the *nScree* function. The resulting maximum number of required PCs (N=8) was used in the *principal* function to extract the rotated PCs. A sphericity test in the *Psych* library [65] confirmed that our PC matrix is significantly different from a spherical matrix (Chi-square=4906, df=253, p<0.001). Furthermore, a sampling adequacy test in the package *REdAS [67]* resulted in a MSA score = 0.82. A MSA score range of 0.8 to 1 indicates adequate sampling, i.e. much fever components relative to the number of variables represent the factors.

Resulting components were interpreted based on the loadings of the original variables as described in detail in Table S2. Observe that including detailed climatic parameters both the entire year and only within the mean growing season reveals detailed insights into the relationship of different climatic parameters. Given that ancestral character reconstruction may be biased when characters influence diversification [68], we also used a phylogenetic controlled PCA [69], resulting is a somewhat different PC structure (Table S4). However, running our main model with this PC resulted qualitatively in the same results (Table S5).

**Generalized linear models**

We fitted phylogenetically controlled multinomial models to our data using MCMCglmm [70]. All models fitted in MCMCglmm were specified using the multinomial distribution family and were run for 6’500’000 iterations, with a 500’000 burn-in period and samples drawn every 500 iterations. The response variable in all models was a categorical representation of social system. Each model included appropriate set of fixed effects (see Results) and a random phylogenetic effect linked to the phylogenetic correlation matrix. Residual covariance matrices in our models were fixed to **IJ** where **I** and **J** are identity and unit matrices, respectively, of dimension equal to the number of response variable categories in the model. Prior covariance matrices for fixed effects where set as (**IJ** x **I_n_**) x (1.7+π^2^/3), where n equals the number of model fixed parameters [71,72]. All results are presented as means of estimated posterior distributions of parameters. Statistical significance of fixed effects was determined based on the pMCMC values returned by MCMCglmm. They represent proportions of all recorded MCMC iterations that lie outside the 95% credibility intervals estimated based on the estimated posterior distributions of parameters.

The phylogenetic random effect was modelled based on a recent phyla-wide phylogeny [62]. For initial and complementary analyses, we used a consensus tree based on the Hackett backbone [73], however we confirmed that using the alternative Ericsson backbone introduces negligible variation to the model estimates (both not shown but available upon request). To account for the uncertainty of phylogeny estimation we refitted the main model with 50 randomly selected trees from the posterior distribution of trees published in Jetz et al. [62]. This number of trees used in our study is sufficient to account for uncertainty in the phylogeny estimation [74]. To summarize the effect of this analysis we calculated the proportion of all 50 models that returns a statistically significant (pMCMC<0.05) effect.

**References**

1. Cockburn A. Prevalence of different modes of parental care in birds. Proceedings of the Royal Society B-Biological Sciences. 2006;273(1592):1375-83. doi: 10.1098/rspb.2005.3458. PubMed PMID: ISI:000237780100010.

2. Russell EM. Avian life histories: Is extended parental care the southern secret? Emu. 2000;100:377-99. PubMed PMID: ISI:000167845800005.

3. Riehl C. Evolutionary routes to non-kin cooperative breeding in birds. Proceedings of the Royal Society B: Biological Sciences. 2013;280(1772):20132245.

4. Cramp S, Simmons KE, Perrins CM, Brooks DJ. Handbook of the birds of Europe, the Middle East and North Africa. Oxford: Oxford University Press; 1994.

5. Del Hoyo J, Elliot A, Sargatal J, Christie DA. Handbook of the Birds of the World. Barcelona: Lynx Editions. 2011.

6. Higgins PJ, Marchant S, Peter JM, Cowling S, Davies J. Handbook of Australian, New Zealand & Antarctic Birds: Oxford University Press; 2007.

7. Hockey PAR, Dean WJJ, Ryan PG. Roberts Birds of Southern Africa. 7th edition ed. Cape Town: Trustees of the John Voelcker Bird Book Fund; 2005.

8. Poole A. The birds of North America online. Cornell Laboratory of Ornithology, Ithaca, NY Available via <http://bnabirdscornelledu/BNA>. 2005.

9. Ali S, Ripley SD. Handbook of the birds of India and Pakistan, together with those of Bangladesh, Nepal, Bhutan, and Sri Lanka. 2002.

10. Fry CH, Fry K. Kingfishers, bee-eaters and rollers: A&C Black; 2010.

11. Madge S. Crows and jays: A&C Black; 2010.

12. Erritzøe J, Mann CF, Brammer FP, Fuller RA. Cuckoos of the world: Bloomsbury (Christopher Helm); 2012.

13. Clement P, Harris A, Davies J. Finches and Sparrows: Bloomsbury (Christopher Helm); 2011.

14. Kennerley P, Pearson D. Reed and bush warblers: A&C Black; 2010.

15. König C, Weick F, Becking J-H. Owls of the world: A&C Black; 2009.

16. Olsen KM. Gulls of Europe, Asia and North America: A&C Black; 2010.

17. Alström P, Mild K, Zetterström B. Pipits and Wagtails of Europe, Asia and North America: A&C Black; 2003.

18. Mann CF, Cheke RA. Sunbirds: a guide to the sunbirds, flowerpeckers, spiderhunters and sugarbirds of the world: A&C Black; 2010.

19. Brewer D, MacKay BK. Wrens, dippers and thrashers: A&C Black; 2001.

20. Ferguson-Lees J, Christie DA, Burton P, Franklin K, Mead D. Raptors of the world: Christopher Helm; 2001.

21. Harris T, Franklin K. Shrikes & bush-shrikes: including wood-shrikes, helmet-shrikes, flycatcher-shrikes, philentomas, batises and wattle-eyes: Christpher Helm; 2000.

22. Isler ML, Isler PR. The tanagers: natural history, distribution, and identification: Christopher Helm; 1987.

23. Jaramillo A, Burke P. New world blackbirds: Christopher Helm; 1999.

24. Feare C, Craig A. Starlings and mynas: Christopher Helm; 1998.

25. Baker K. Warblers of Europe, Asia, and North Africa: Christopher Helm; 1997.

26. Harrap S, Quinn D. Tits, Nuthatches and Treecreepers: An Identification Guide. London: Christopher Helm; 1995.

27. Kear J. Ducks, Geese and Swans: Anseriformes. Oxford: Oxford University Press; 2005.

28. Kemp AC, Woodcock M. The hornbills: bucerotiformes: Oxford University Press Oxford; 1995.

29. Forshaw JM. Parrots of the World: Princeton University Press; 2010.

30. Onley D, Scofield P. Albatrosses, petrels and shearwaters of the world: A&C Black; 2007.

31. Drobniak SM, Wagner G, Mourocq E, Griesser M. Family living: an overlooked but pivotal social system to understand the evolution of cooperative breeding. Behavioral Ecology. 2015;26(3):805-11. doi: 10.1093/beheco/arv015.

32. Jetz W, Rubenstein DR. Environmental Uncertainty and the Global Biogeography of Cooperative Breeding in Birds. Current Biology. 2011;21(1):72-8. doi: 10.1016/j.cub.2010.11.075. PubMed PMID: WOS:000286485200029.

33. du Plessis MA, Siegfried WR, Armstrong AJ. Ecological and Life-History Correlates of Cooperative Breeding in South-African Birds. Oecologia. 1995;102(2):180-8. PubMed PMID: ISI:A1995QY66400008.

34. Ford HA, Bell H, Nias R, Noske R. The Relationship between Ecology and the Incidence of Cooperative Breeding in Australian Birds. Behavioral Ecology and Sociobiology. 1988;22(4):239-49. PubMed PMID: ISI:A1988M990200002.

35. Arnold KE, Owens IPF. Cooperative breeding in birds: a comparative test of the life history hypothesis. Proceedings of the Royal Society B-Biological Sciences. 1998;265(1398):739-45. PubMed PMID: ISI:000073580200001.

36. Cockburn A. Cooperative breeding in oscine passerines: does sociality inhibit speciation? Proceedings of the Royal Society B-Biological Sciences. 2003;270(1530):2207-14. PubMed PMID: ISI:000186630800002.

37. Griesser M, Suzuki TN. Occasional cooperative breeding in birds and the robustness of comparative analyses concerning the evolution of cooperative breeding. Zoological Letters. 2016:2:7.

38. Baglione V, Marcos JM, Canestrari D, Griesser M, Andreotti G, Bardini C, et al. Does year-round territoriality rather than habitat saturation explain delayed natal dispersal and cooperative breeding in the carrion crow? Journal of Animal Ecology. 2005;74(5):842-51. PubMed PMID: ISI:000231612100005.

39. Gayou DC. The Social System of the Texas Green Jay. Auk. 1986;103(3):540-7. PubMed PMID: ISI:A1986D200300010.

40. Cockburn A. Why do so many Australian birds cooperate: social evolution in the Corvida? In: Floyd RB, Sheppard AW, Barro PJD, editors. Frontiers of population ecology. Melbourne: CSIRO; 1996. p. 451-72.

41. Selander RK. Speciation in the wrens of the genus Campylorhynchus. University of California Publications in Zoology. 1964;74:1-224.

42. Brown JL. Helping and Communal Breeding in Birds. Princeton: Princeton University Press; 1987.

43. Emlen ST. The Evolution of Helping .1. an Ecological Constraints Model. American Naturalist. 1982;119(1):29-39. PubMed PMID: ISI:A1982MY09300003.

44. Ekman J, Baglione V, Eggers S, Griesser M. Delayed dispersal: Living under the reign of nepotistic parents. Auk. 2001;118(1):1-10. PubMed PMID: ISI:000167402400001.

45. Covas R, Griesser M. Life history and the evolution of family living in birds. Proceedings of the Royal Society B-Biological Sciences. 2007;274(1616):1349-57. PubMed PMID: WOS:000246478500001.

46. Ekman J, Rosander B. Survival Enhancement Through Food Sharing - A Means For Parental Control Of Natal Dispersal. Theoretical Population Biology. 1992;42(2):117-29. PubMed PMID: ISI:A1992JZ81400002.

47. IUCN. IUCN Habitat Classification Scheme2007 accessed March 2010.

48. Mueller-Dombois D, Ellenberg H. Aims and methods of vegetation ecology. new York: Wiley & Sons, Inc.; 1974.

49. Dunning JB. Body weights of 686 species of North American birds. West Bird Banding Association Monogr# 1, Cave Creek, Arizona ii+ 38 pp. 1984.

50. Starck JM, Ricklefs RE. Avian growth and development: evolution within the altricial-precocial spectrum: Oxford University Press; 1998.

51. Valcu M, Dale J, Griesser M, Nakagawa S, Kempenaers B. Global gradients of avian longevity support the classic evolutionary theory of ageing. Ecography. 2014;37(10):930-8. doi: 10.1111/ecog.00929.

52. ABBBS. ABBBS database. <http://www.environment.gov.au/science/bird-and-bat-banding/banding-data:> Australian Bird and Bat Banding Scheme; 2014.

53. Young AM, Hobson EA, Lackey LB, Wright TF. Survival on the ark: life‐history trends in captive parrots. Animal conservation. 2012;15(1):28-43.

54. Jones P, Harris I. University of East Anglia Climatic Research Unit, CRU TS3. 21: Climatic Research Unit (CRU) Time-Series (TS) Version 3.21 of High Resolution Gridded Data of Month-by-month Variation in Climate (Jan. 1901—Dec. 2012). NCAS British Atmospheric Data Centre. 2013.

55. Justice CO, Vermote E, Townshend JR, Defries R, Roy DP, Hall DK, et al. The Moderate Resolution Imaging Spectroradiometer (MODIS): Land remote sensing for global change research. Geoscience and Remote Sensing, IEEE Transactions on. 1998;36(4):1228-49.

56. Colwell RK. Predictability, Constancy, and Contingency of Periodic Phenomena. Ecology. 1974;55(5):1148-53. PubMed PMID: ISI:A1974U731500022.

57. Rubenstein DR, Lovette IJ. Temporal environmental variability drives the evolution of cooperative breeding in birds. Current Biology. 2007;17(16):1414-9. PubMed PMID: WOS:000248989500037.

58. Morison JI, Morecroft MD. Plant growth and climate change: John Wiley & Sons; 2008.

59. Botero CA, Dor R, McCain CM, Safran RJ. Environmental harshness is positively correlated with intraspecific divergence in mammals and birds. Molecular ecology. 2014;23(2):259-68.

60. R Developement Core Team. R: A language and environment for statistical computing. ISBN 3-900051-07-0. R Foundation for Statistical Computing. Vienna, Austria, 2013. url: <http://www.R-project.org>, 2013.

61. FitzJohn RG. Diversitree: comparative phylogenetic analyses of diversification in R. Methods in Ecology and Evolution. 2012;3(6):1084-92.

62. Jetz W, Thomas GH, Joy JB, Hartmann K, Mooers AO. The global diversity of birds in space and time. Nature. 2012;491(7424):444-8. doi: 10.1038/nature11631.

63. Akaike H. New Look at Statistical-Model Identification. Ieee Transactions on Automatic Control. 1974;AC19(6):716-23. PubMed PMID: ISI:A1974U921700011.

64. Revell LJ. phytools: an R package for phylogenetic comparative biology (and other things). Methods in Ecology and Evolution. 2012;3(2):217-23.

65. Revelle W. Procedures for psychological, psychometric, and personality research. Computer software manual] Evanston, IL: Northwestern University Retrieved from <http://wwwpersonality-project> net/r/psychmanual pdf (R package version 13 2). 2012.

66. Raiche G, Magis D. nFactors: Parallel analysis and non graphical solutions to the Cattell Scree Test. R package version. 2010;2(3).

67. Maier MJ, Maier MMJ. Package ‘REdaS’. 2015.

68. Maddison WP. Confounding asymmetries in evolutionary diversification and character change. Evolution. 2006;60(8):1743-6.

69. Revell LJ. Size‐correction and principal components for interspecific comparative studies. Evolution. 2009;63(12):3258-68.

70. Hadfield JD. MCMC methods for multi-response generalized linear mixed models: the MCMCglmm R package. Journal of Statistical Software. 2010;33(2):1-22.

71. Hadfield J. MCMCglmm course notes2014.

72. Hadfield JD, Nakagawa S. General quantitative genetic methods for comparative biology: phylogenies, taxonomies and multi-trait models for continuous and categorical characters. J Evol Biol. 2010;23(3):494-508. doi: 10.1111/j.1420-9101.2009.01915.x.

73. Hackett SJ, Kimball RT, Reddy S, Bowie RCK, Braun EL, Braun MJ, et al. A Phylogenomic Study of Birds Reveals Their Evolutionary History. Science. 2008;320(5884):1763-8. doi: 10.1126/science.1157704.

74. Nakagawa S, de Villemereuil P. A simple and general method for accounting for phylogenetic uncertainty via Rubin’s rules in comparative analysis. PeerJ PrePrints. 2015;3:e1488.
